# Transient IKK2 activation in astrocytes initiates selective non-cell-autonomous neurodegeneration

**DOI:** 10.1186/s13024-017-0157-0

**Published:** 2017-02-13

**Authors:** Michael Lattke, Stephanie N. Reichel, Alexander Magnutzki, Alireza Abaei, Volker Rasche, Paul Walther, Dinis P. Calado, Boris Ferger, Thomas Wirth, Bernd Baumann

**Affiliations:** 1 Institute of Physiological Chemistry, Ulm University, Albert-Einstein-Allee 11, 89081 Ulm, Germany; 2 Core Facility Small Animal MRI, Ulm University, Albert-Einstein-Allee 11, 89081 Ulm, Germany; 3 Central Facility for Electron Microscopy, Ulm University, Albert-Einstein-Allee 11, 89081 Ulm, Germany; 4 Immunity and Cancer Laboratory, The Francis Crick Institute, 1 Midland Road, London, NW1 1AT UK; 5 CNS Diseases Research, Boehringer Ingelheim Pharma GmbH & Co. KG, Birkendorfer Str. 65, 88397 Biberach an der Riss, Germany; 6 Neural Stem Cell Biology Laboratory, The Francis Crick Institute, 1 Midland Road, London, NW1 1AT UK

**Keywords:** NF-kappaB, IKK, Neuroinflammation, Cerebellar ataxia, Purkinje cell degeneration, Bergmann glia, Glutamate transporter, EAAT, Excitotoxicity

## Abstract

**Background:**

Neuroinflammation is associated with a wide range of neurodegenerative disorders, however the specific contribution to individual disease pathogenesis and selective neuronal cell death is not well understood. Inflammatory cerebellar ataxias are neurodegenerative diseases occurring in various autoimmune/inflammatory conditions, e.g. paraneoplastic syndromes. However, how inflammatory insults can cause selective cerebellar neurodegeneration in the context of these diseases remains open, and appropriate animal models are lacking. A key regulator of neuroinflammatory processes is the NF-κB signalling pathway, which is activated by the IκB kinase 2 (IKK2) in response to various pathological conditions. Importantly, its activation is sufficient to initiate neuroinflammation on its own.

**Methods:**

To investigate the contribution of IKK/NF-κB-mediated neuroinflammation to neurodegeneration, we established conditional mouse models of cerebellar neuroinflammation, which depend either on the tetracycline-regulated expression of IKK2 in astrocytes or Cre-recombination based IKK2 activation in Bergmann glia.

**Results:**

We demonstrate that IKK2 activation for a limited time interval in astrocytes is sufficient to induce neuroinflammation, astrogliosis and loss of Purkinje neurons, resembling the pathogenesis of inflammatory cerebellar ataxias. We identified IKK2-driven irreversible dysfunction of Bergmann glia as critical pathogenic event resulting in Purkinje cell loss. This was independent of Lipocalin 2, an acute phase protein secreted by reactive astrocytes and well known to mediate neurotoxicity. Instead, downregulation of the glutamate transporters EAAT1 and EAAT2 and ultrastructural alterations suggest an excitotoxic mechanism of Purkinje cell degeneration.

**Conclusions:**

Our results suggest a novel pathogenic mechanism how diverse inflammatory insults can cause inflammation/autoimmune-associated cerebellar ataxias. Disease-mediated elevation of danger signals like TLR ligands and inflammatory cytokines in the cerebellum activates IKK2/NF-κB signalling in astrocytes, which as a consequence triggers astrogliosis-like activation of Bergmann glia and subsequent non-cell-autonomous Purkinje cell degeneration. Notably, the identified hit and run mechanism indicates only an early window for therapeutic interventions.

**Electronic supplementary material:**

The online version of this article (doi:10.1186/s13024-017-0157-0) contains supplementary material, which is available to authorized users.

## Background

Neuroinflammation is found in many neurological disorders, including Alzheimer’s disease and autoimmune diseases like multiple sclerosis and paraneoplastic syndromes [1, 2]. Despite intensive research, the contribution of neuroinflammation to the pathogenesis of these disorders and underlying mechanisms accounting for selective vulnerability of specific neuronal populations remain poorly understood.

During neuroinflammation, both immune cells and neural cells respond to and produce diverse mediators, creating a complex cross-talk which can culminate in either neuroprotection or neurodegeneration [2]. Astrocytes are key players in this neuro-immune crosstalk as they detect and respond to various alterations of CNS homeostasis. In response to pathological conditions astrocytes start to express proinflammatory factors that mediate recruitment and activation of immune cells [3]. This activation process called astrogliosis is characterized by astrocyte hypertrophy, proliferation, and enhanced expression of the astroglial intermediate filament GFAP. It also alters the homeostatic functions of astrocytes, like the uptake of glutamate to prevent neurotoxic extracellular accumulation [4]. Although astrogliosis can have beneficial functions, it often impairs neural homeostasis and regeneration after injury [4].

Astrocyte activation can be mediated by a large repertoire of receptors that induce various signalling pathways, including the IKK/NF-κB system, a master regulator of inflammation [5]. NF-κB is activated by proinflammatory stimuli like TNFα, IL-1β or TLR ligands and regulates a large number of genes involved in inflammation, proliferation, cell survival, and specific CNS functions [6, 7]. Most NF-κB activating pathways converge in the activation of the IκB kinase (IKK) complex, with IKK2 as crucial kinase subunit that induces degradation of NF-κB inhibitor proteins of the IκB family. This results in the nuclear translocation of NF-κB dimers, where they regulate transcription of target genes [8].

Since NF-κB activation is a key step in inflammatory responses and astrocytes play a prominent role in such processes in the CNS, the functional investigation of IKK/NF-κB signalling in astrocytes is important for the understanding and treatment of disorders associated with neuroinflammation. Repression of NF-κB activation in astrocytes reduced neuroinflammation in spinal cord injury, retinal ischemia reperfusion injury, and autoimmune encephalitis [9–13]. We have recently shown that genetic IKK2/NF-κB activation in astrocytes is sufficient to initiate neuroinflammation, resulting in developmental hydrocephalus formation [14].

Here, we take advantage of this conditional model to investigate the consequences of neuroinflammation in adult mice on cerebellar homeostasis. We provide evidence that astrocyte-driven neuroinflammation in the cerebellum induces selective, non-cell-autonomous degeneration of Purkinje neurons. This process is triggered by activation-induced dysfunction of Bergmann glia, a specialized astrocyte population in the cerebellum.

## Results

### IKK2-mediated chronic neuroinflammation results in progressive cerebellar atrophy and ataxia

**Fig. 1:**
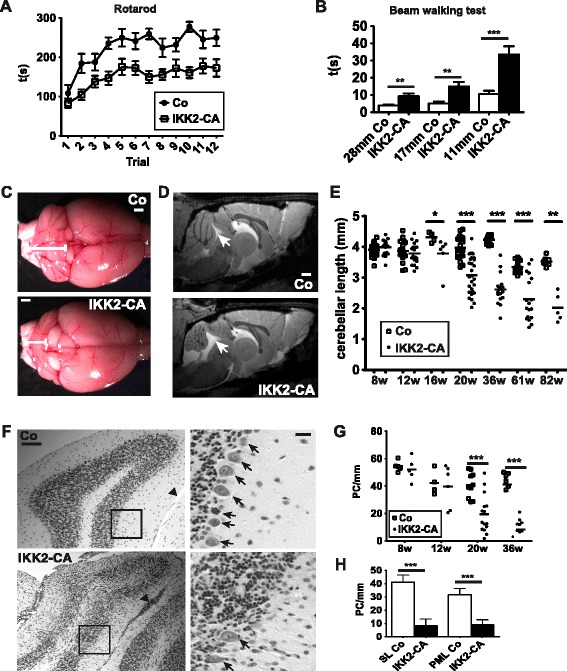
Expression of constitutively active IKK2 in astrocytes causes cerebellar atrophy and ataxia characterized by prominent Purkinje cell loss. **a** Fast motor coordination is impaired in IKK2-CA mice at the age of 30-34 weeks. Latency to fall off an accelerating rotarod is reduced. Mean values +/- s.e.m.; statistical analysis: 2-way-ANOVA (*n* = 11–15), *p* < 0.0001. **b** Impaired balance/movement precision in IKK2-CA mice at 30–34 weeks as determined by time required to cross a narrow beam, diameter 11/17/28 mm. mean values +/- s.e.m.; statistical analysis: 2-tailed unpaired *t*-test (*n* = 11–15), ** *p* < 0.01; *** *p* < 0.001. **c** IKK2-CA expression results in macroscopic cerebellar atrophy at 36 weeks. Scale bar: 1 mm. **d** Cerebellar atrophy at 50 weeks shown by MRI (sagittal T2*-weighed image at the midline). *Arrow*: enlarged CSF filled ventricular cavity due to the reduced cerebellar volume. Scale bar: 1 mm. **e** Variable onset of cerebellar atrophy between 12 and 20 weeks of age and further progression until the age of 82 weeks. Diagram shows maximal rostro-caudal length of the cerebellum (single animals and mean); statistical analysis: 2-tailed Mann-Whitney-test, * *p* < 0.05; ** *p* < 0.01; *** *p* < 0.001. **f** Histological analysis of the simple lobule reveals loss of Purkinje cells in the IKK2-CA model. *Arrowhead*: cells in meningeal foldings, *arrows*: Purkinje cells. Scale bars: 100 μm (*left panels*); 20 μm (enlargements, *right panels*). **g** Time course of Purkinje cell loss in the simple lobule. Quantification from Nissl stainings. Statistical analysis: 2-tailed unpaired *t*-test, *** *p* < 0.001, other time points *p* > 0.05. **h** Purkinje cell loss in the simple lobule (SL) and the paramedian lobule (PML) at 36 weeks of age; statistical analysis: 2-tailed unpaired *t*-test (*n* = 6–8), *** *p* < 0.001

To investigate the impact of neuroinflammation on CNS homeostasis in the adult mouse, we used a previously established conditional mouse model that allows expression of a constitutively active IKK2 allele in astrocytes in a doxycycline-dependent manner [14]. This gain-of-function mouse model (GFAP.tTA/tetO.IKK2-CA, abbreviated IKK2-CA from hereon) is characterized by a robust neuroinflammatory phenotype with lethal hydrocephalus formation in early postnatal development [14]. When IKK2-CA transgene expression is switched off during early postnatal development by doxycycline administration and activated later by doxycycline withdrawal at the age of 4 weeks, animals also develop neuroinflammation [14, 15] that initially results only in transient, usually mild signs of sickness (data not shown). However, after permanent transgene expression until the age of 7–8 months, animals displayed severe ataxia-like motor impairments in rotarod and beam-walking tests (Fig. 1a and b).

The main control centre for motor coordination is the cerebellum, which displayed severe atrophy at 36 weeks of age (Fig. 1c). MRI analysis at 50 weeks confirmed the severe cerebellar atrophy, whereas it did not reveal any obvious alterations in other brain regions (Fig. 1d). Measurement of the rostro-caudal length of the cerebellum in different age groups showed an onset of atrophy around the age of 12 weeks (Fig. 1e). At 20 weeks the majority of animals display severe atrophy indicating rapid progression of degeneration (Fig. 1e). Remarkably, motor deficits are already present at 10 weeks, indicating earlier subtle cerebellar deficits preceding macroscopic atrophy (Additional file 1: Figure S1A).

Nissl staining did not reveal cerebellar alterations at the age of 8 weeks, i.e. before onset of macroscopic atrophy, but a prominent compression of the cerebellar cortex, especially the molecular layer, was observed at 36 weeks (Additional file 1: Figure S1B). This was associated with a prominent loss of Purkinje neurons, accumulation of cells in the meningeal zone and in severe cases also with a loss of cells in the internal granule layer (Fig. 1f). Purkinje cell loss at different ages (8–36 weeks) in the simple lobule correlated with the time course of cerebellar atrophy (Fig. 1g). The extent of degeneration is comparable throughout the cerebellum, e.g. the paramedian lobule shows cell loss similar to the simple lobule (Fig. 1h).

When we expressed IKK2-DN, a kinase inactive allele of IKK2 [16], in astrocytes, no cerebellar degeneration was observed (Additional file 1: Figure S1C and D) indicating that the phenotype is dependent on IKK2 kinase activity.

### Cerebellar neuroinflammation involves local glial responses and infiltration of peripheral immune cells

**Fig. 2:**
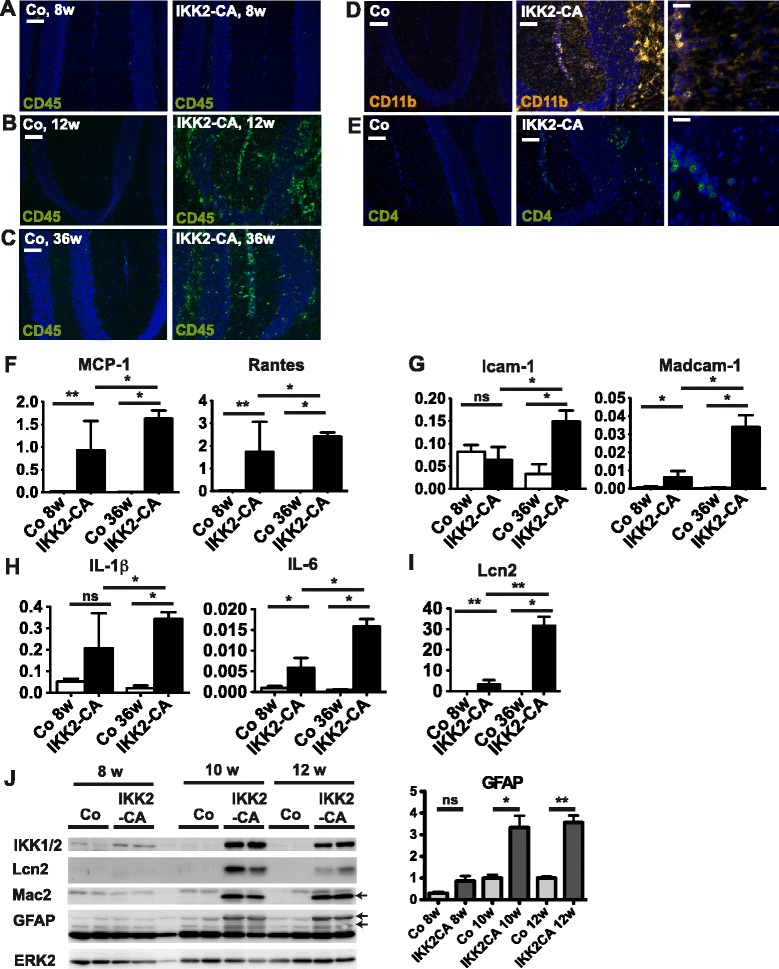
IKK2-CA animals exhibit prominent cerebellar neuroinflammation including microgliosis and astrogliosis. **a**-**c** Activation/infiltration of CD45 positive immune cells at different ages (8–36 weeks) in the cerebellum of IKK2-CA animals. **d** Staining for the myeloid cell marker CD11b indicates massive microgliosis at 12 weeks of age. Higher magnification (*right panels*): microglia display arborized morphology; highly activated microglia/infiltrated macrophages are rounded (amoeboid) cells. **e** Infiltration of Th-cells, shown by CD4 staining (age 12 weeks); right panels: higher magnification. **f**-**i** IKK2-CA induces a proinflammatory gene expression profile in the cerebellum of IKK2-CA mice increasing with age (qRT-PCR). Expression of chemokines (**f**), cell adhesion molecules (**g**), inflammatory cytokines (**h**) and the acute phase response factor Lcn2 (**i**) is elevated in IKK2-CA mice. Expression levels presented relative to HPRT (mean +/- s.e.m.); statistical analysis: 2-tailed Mann-Whitney-test (*n* = 4–7), ns: not significant (*p* > 0.05); * *p* < 0.05; ** *p* < 0.01. **j** Immunoblotting for Mac2 and GFAP indicates microgliosis and astrogliosis respectively, transgene expression (IKK1/2 immunoreactivity) and induction of the NF-κB target Lcn2. Representative immunoblot and quantification of GFAP immunoreactivity normalized to ERK2 (loading control), shown as mean +/- s.e.m. relative to Co 10w, statistical analysis: 2-tailed unpaired *t*-test (*n* = 3–4), ns: not significant; * *p* < 0.05; ** *p* < 0.01; *** *p* < 0.001. Images (**a**-**e**) show DAPI co-staining. Scale bars: (**a**-**c**) 100 μm; (**d**-**e**) 100 μm, right panels 25 μm

To address the mechanisms leading to selective cerebellar neurodegeneration we performed a detailed analysis of the inflammatory response elicited by IKK2 activation in astrocytes. At the age of 8 weeks when there is no obvious cerebellar degeneration, we did not observe significant inflammatory infiltration in the cerebellum (Fig. 2a). However, a prominent increase in CD45 immunoreactivity was detected from 12 weeks (Fig. 2b) up to at least 36 weeks of age (Fig. 2c), indicating persistent neuroinflammation. The myeloid marker CD11b (Fig. 2d) and the macrophage/microglia marker Mac-2 (Additional file 1: Figure S2A) revealed a prominent increase in immunoreactivity, mainly of cells of arborized/ramified microglial morphology, indicating strong microgliosis. We did not observe any infiltration of B-cells by B220 and CD19 staining (data not shown) but could detect limited numbers of CD4- and CD8-positive T-cells (Fig. 2e and Additional file 1: Figure S2B).

The subsequent characterization of the inflammatory gene expression profile demonstrated that already at the age of 8 weeks, before detectable immune cell infiltration, astrocyte-specific IKK2 activation is sufficient to initiate a gene expression pattern correlating with the observed innate immune response (microgliosis) and adaptive immune cell recruitment (T-cell infiltration). Similar to the profile identified at early postnatal development and in primary astrocytes [14] we found diverse NF-κB target genes upregulated, including chemokines and cell adhesion molecules, which are involved in immune cell recruitment, specifically MCP-1, Rantes, Icam-1, Madcam-1 (Fig. 2f and g), as well as the cytokines IL-1β and IL-6 (Fig. 2h) implicated in their activation. Also indicative of immune cell activation, immune effector genes were induced, such as MHC class II proteins and complement, specifically CD74, H2-Aa, C3, C4b (Additional file 1: Figure S2C and D) and the acute phase response factor and NF-κB target gene Lcn2 (Fig. 2i). Expression of these factors further increased towards a late stage of cerebellar degeneration (Fig. 2f-i; Additional file 1: Figure S2C and D). Expression of Lcn2 protein from 10 weeks onwards was accompanied by upregulation of GFAP and Mac2 as markers of astrogliosis and microgliosis (Fig. 2j). These data show that 6–8 weeks after doxycycline withdrawal, when IKK2-CA transgene expression reaches its full extent (Fig. 2j), a full-blown neuroinflammatory response is elicited in the cerebellum of IKK2-CA mice.

Remarkably, prominent infiltration of CD45 positive cells is also found in various other brain regions (Additional file 1: Figure S3A-D), demonstrating that IKK2-mediated neuroinflammation is not restricted to the cerebellum. However, even after long-term IKK2 activation, at 36 weeks of age, no neurodegeneration is detected in other brain regions. Nissl staining does not reveal any obvious atrophy or cell loss in any brain region beside the cerebellum (Additional file 1: Figure S3E), and the quantification of NeuN positive neurons in the medulla oblongata, another region with prominent neuroinflammation (Additional file 1: Figure S3D), does not indicate any neuronal loss (Additional file 1: Figure S3F and G).

These data show that genetic IKK2 activation in astrocytes induces global neuroinflammation, likely by the induction of various proinflammatory factors, in line with its function as central activator of the NF-κB signalling pathway. However, neurodegeneration is restricted to the cerebellum, primarily to Purkinje cells, indicating a selective vulnerability of Purkinje cells to neuroinflammation and astroglial IKK2 activation.

### Purkinje cell loss progresses independent of IKK2 activation and neuroinflammation after onset of ataxia

**Fig. 3:**
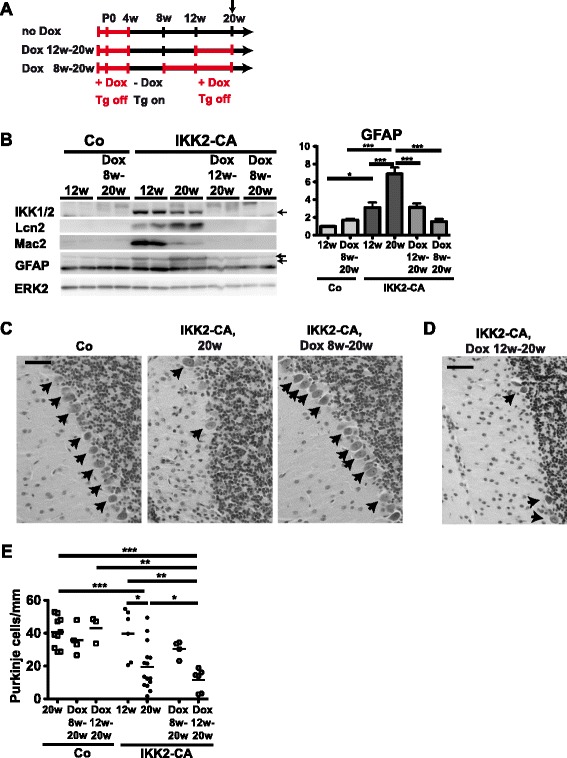
IKK2-CA inactivation after onset of ataxia abrogates neuroinflammation and arrests astrogliosis, but cannot prevent Purkinje cell loss. **a** Schedule of doxycycline treatment to turn off IKK2-CA expression at different stages of phenotype development. No Dox: continuous expression starting at 4 weeks of age, Inactivation of expression before onset of ataxia (Dox 8–20 weeks) or before onset of substantial Purkinje cell loss (Dox 12–20 weeks). **b** Immunoblotting for Mac-2 and GFAP, indicating microgliosis and astrogliosis. Lcn2 indicates NF-κB activation, IKK1/2 immunoreactivity IKK2-CA expression. Representative immunoblot and quantification of GFAP immunoreactivity normalized to ERK2 (loading control), shown as mean +/- s.e.m. relative to Co 12w (*n* = 3–4). **c**-**e** Purkinje cell loss at 20 weeks (compared to controls) is prevented by early repression of IKK2-CA at 8 weeks (**c**), but not by repression at 12 weeks of age (**d**). Representative Nissl staining (**c**, **d**) and quantification (**e**) is depicted. Values of untreated animals are also presented in Fig. 1e. Scale bars 50 μm. Statistical analysis (**b**, **e**): 1-way ANOVA with Tukey’s post-test, * *p* < 0.05; ** *p* < 0.01; *** *p* < 0.001

We next asked whether neurodegeneration depends on continuous IKK2 activation and chronic neuroinflammation or whether it follows a hit-and-run mechanism. For this purpose, we inactivated IKK2-CA expression by administration of doxycycline in two experimental settings (Fig. 3a), either at the pre-symptomatic stage (8 weeks of age), or early after the onset of ataxia and inflammation, but before substantial Purkinje cell loss (12 weeks of age). Animals with continuous IKK2-CA expression starting at 4 weeks of age show prominent inflammation and astrogliosis at 20 weeks of age, indicated by Lcn2, Mac2 and GFAP expression, respectively (Fig. 3b) that correlates with prominent Purkinje cell loss (Fig. 3c and e). In contrast, when transgene expression is inactivated at 8 weeks, animals show neither Purkinje cell loss (Fig. 3c and e) nor elevated levels of Lcn2, Mac2 or GFAP (Fig. 3b). Notably, when transgene expression was inactivated at 12 weeks, Purkinje cell loss continued despite normalized levels of Lcn2 and Mac2 and an arrest of astrogliosis indicated by GFAP immunoreactivity (Fig. 3b, d and e). Importantly, this continuing Purkinje cell degeneration is likely not due to a slow transgene inactivation or delayed resolution of inflammation, as already 2 weeks after doxycycline re-administration (Dox 12w-14w, Additional file 1: Figure S4A) transgene, Lcn2 and Mac2 expression is almost undetectable (Additional file 1: Figure S4B and C). In contrast, GFAP levels indicative for astrogliosis remain elevated (Additional file 1: Figure S4B and C). Furthermore, various IKK2-CA-induced inflammatory mediators, including the major proinflammatory cytokines TNFα, IL-1β, Rantes (CCL5) and IL-6, have returned close to control levels (Additional file 1: Figure S4D), with the exception of MCP-1 (CCL2) and Icam-1 (Additional file 1: Figure S4E), indicating that neuroinflammation is already largely resolved at this early time point. Together these results demonstrate that IKK2 activation in astrocytes for a limited time interval is able to trigger Purkinje cell degeneration, which after this initial hit becomes independent of IKK2 activation and neuroinflammation.

### Purkinje cell loss is caused by irreversible Bergmann glia dysfunction

The Bergmann glia is a specialized radial glia-like astrocyte subtype in the cerebellar cortex, which displays a unique interaction with Purkinje cells. The cell bodies of Bergmann glia are located in the Purkinje cell layer and extend radially arranged Bergmann glia fibres that enwrap synapses on Purkinje cell dendrites. This tight association is important for Purkinje cell survival [17–20] and we asked whether astroglial IKK2 activation disrupts this interaction and thereby induces the prominent Purkinje cell degeneration.

**Fig. 4:**
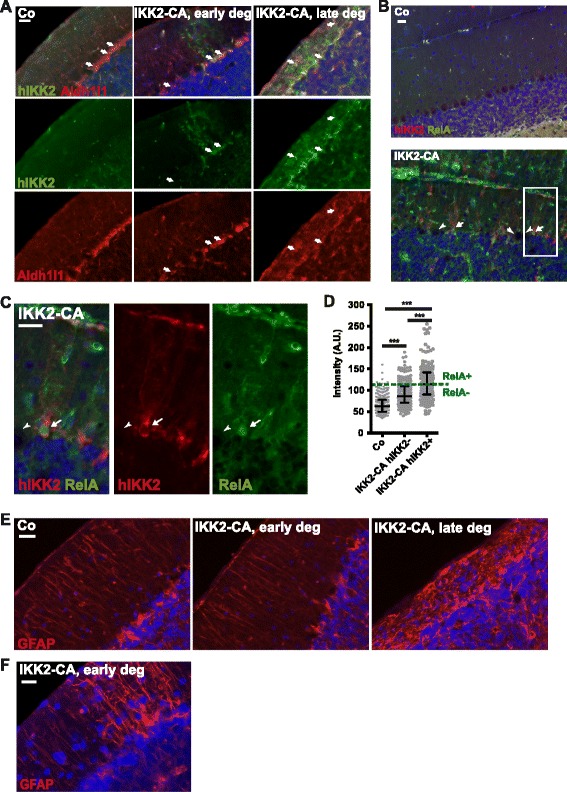
IKK2-CA expression in Bergmann glia disrupts their morphology in correlation with the stage of cerebellar degeneration. **a** Co-staining for hIKK2 (transgene) and the astrocyte marker Aldh1l1 at 20 weeks of age in a cerebellum without detectable degeneration (“early deg”) and in a late stage of degeneration (“late deg”) Arrows: Bergmann glia cell bodies. **b**-**d** IKK2-CA-expressing (hIKK2 positive) Bergmann glia marked by arrows display nuclear localization of RelA indicating active NF-κB signalling (**b**). Arrowheads: Purkinje cell bodies (hIKK2 negative). **c** Enlargement of the box in B. **d** Quantification of nuclear RelA immunofluorescence intensity in cells in the Purkinje cell layer, including IKK2-CA (hIKK2) expressing Bergmann glia. *n* = 360 cells of 3 control and *n* = 480 of 4 IKK2-CA animals. Statistical analysis: 1-way ANOVA with Tukey’s post-test, *** *p* < 0.001. **e** Staining for GFAP at 20 weeks of age shows Bergmann glia processes in the molecular layer. Controls and IKK2-CA cerebella without detectable degeneration (“early deg”) show parallel Bergmann glia processes with weak GFAP. Severely degenerated cerebella (“late deg”) show intensely stained, thickened and unorganized processes. **f** Occasionally, IKK2-CA animals without obvious Purkinje cell loss (age 12 weeks) show patches of Bergmann glia with increased GFAP expression and first signs of disorganisation. Merged images show DAPI co-staining (*blue*). Scale bars: 20 μm

We could confirm that the IKK2-CA transgene is expressed in Bergmann glia, but not in Purkinje cells and microglia by co-staining analyses using an antibody recognizing only the human IKK2 derived transgene together with the astrocyte marker Aldh1l1 (Fig. 4a), the Purkinje cell marker Calbindin (Additional file 1: Figure S5A) or the microglia marker Iba1 (Additional file 1: Figure S5B). There were a few IKK2-CA mice that do not display obvious degeneration at the age of 20 weeks. These mice show only week occasional IKK2-CA transgene expression in Bergmann glia and an Aldh1l1 staining pattern similar to controls (Fig. 4a, early deg). In animals of the same age, but with prominently degenerated cerebella, strong transgene and Aldh1l1 expression was found mainly in the molecular layer (Fig. 4a, late deg). This indicates that Purkinje cell loss is associated with a distortion of Bergmann glia structure driven by IKK2-CA. Quantitative analysis of the subcellular localisation of the NF-κB subunit RelA in IKK2-CA expressing Bergmann glia by co-immunostaining for RelA and the transgene revealed a prominent increase in nuclear localization of RelA (Fig. 4b-d), demonstrating NF-κB activation in these cells.

The extent of structural alterations of Bergmann glia was confirmed by GFAP staining. In pre-symptomatic cerebella low GFAP containing parallel fibres crossed the molecular layer similar to controls (Fig. 4e). In contrast, Bergmann glia processes in the molecular layer were thicker, disorganized, and more intensely stained in degenerated cerebella (Fig. 4e), indicating astrogliosis-like activation of the Bergmann glia. Interestingly, in IKK2-CA cerebella without obvious degeneration, occasionally individual Bergmann glia could be detected that showed increased expression of GFAP and moderate disorganization (Fig. 4f), suggesting that Bergmann glia activation might precede Purkinje cell loss.

Given that permanent IKK2 activation in astrocytes is not required for progression of degeneration, Purkinje cell loss could be driven by an early hit resulting in irreversible Bergmann glia dysfunction followed by delayed Purkinje cell loss as a consequence. In this scenario, Purkinje cell loss should only occur in areas with activated Bergmann glia. Therefore, repression of IKK2-CA from 12 weeks of age would arrest further Bergmann glia activation, but Purkinje cell loss could further continue in areas where previously Bergmann glia have been activated, thereby explaining progression of degeneration after block of transgene expression.

Alternatively, Purkinje cell loss could be triggered by astrocyte-mediated early Purkinje cell damage (e.g. via neurotoxic factors released by astrocytes) before 12 weeks of age. Here, Bergmann glia activation takes place as a local secondary event caused by neuronal degeneration. In this scenario, areas with Purkinje cell loss would not necessarily show Bergmann glia activation, and the pattern of Purkinje cell loss and Bergmann glia activation would be independent of IKK2-CA inactivation starting at 12 weeks of age.

**Fig. 5:**
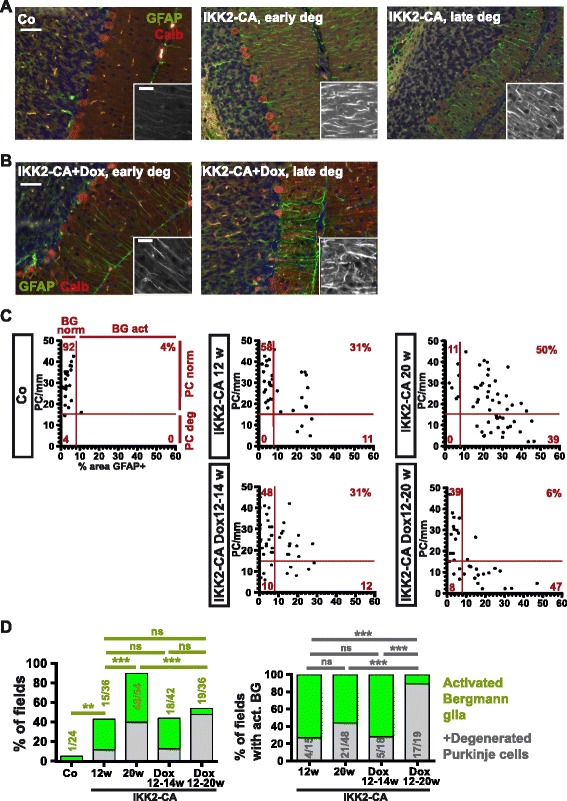
Local IKK2-CA-induced Bergmann glia activation drives subsequent Purkinje cell loss. **a**, **b** Variable loss of Purkinje cells (calbindin-positive) in areas with astrogliosis-like Bergmann glia activation (increased GFAP staining) in IKK2-CA animals at 20 weeks. Areas without detectable degeneration (“early deg”) showing parallel Bergmann glia processes and degenerated areas (“late deg”) are depicted (**a**). After IKK2-CA repression by doxycycline from 12 weeks of age severe Purkinje cell loss at 20 weeks is evident in areas with Bergmann glia activation, but not in fields with normal Bergmann glia (**b**). Inlays: higher magnification of GFAP channel in the molecular layer. DAPI co-staining (*blue*). Scale bars 50 μm (inlays 20 μm). **c**, **d** Quantitative analysis of the spatial correlation of Bergmann glia activation (>8% area GFAP+ in the molecular layer) and Purkinje cell degeneration (<15 Purkinje cells/mm) in individual microscopic fields. Degeneration is almost exclusively found in fields with activated Bergmann glia (**c**). Bergmann glia activation increases between 12 and 20 weeks in IKK2-CA expressing animals, but is arrested by doxycycline (C/D). Fields with activated Bergmann glia display variable Purkinje cell loss at 20 weeks in animals expressing IKK2-CA, whereas after treatment with doxycycline from 12 to 20 weeks, most fields with activated Bergmann glia show prominent Purkinje cell loss (C/D). Purkinje cell loss progresses between 2 weeks (Dox 12-14w) and 8 weeks (Dox 12-20w) after doxycycline application in fields with Bergmann glia activation. **c** Bergmann glia activation and Purkinje cell numbers in individual fields. *Red numbers*: percentage of fields in the specific quadrant. Analysis of 6 fields/animal with *n* = 4/6/9/7/6 animals for Co/IKK2-CA12w/20w/Dox 12-14w/Dox 12-20w. **d** Percentage and absolute numbers of fields with activated Bergmann glia (*green*) and Purkinje cell loss (*grey*) from Co and IKK2-CA mice at the age of 12, 14 and 20 weeks (12w, Dox 12-14w, 20w) are shown. Numbers represent fields with activated Bergmann glia vs. total fields analysed (left panel) and fields with degenerated Purkinje cells and activated Bergmann glia vs. total fields with activated Bergmann glia (*right panel*). Transgene inactivation via doxycycline application at the age of 12 weeks (Dox 12-20w) stops the increase in Bergmann glia activation compared to animals at 12 w (*left panel*), but does not stop Purkinje cell loss (*right panel*). Statistical analysis: Fisher’s exact test, ns: not significant (*p* > 0.05); ** *p* < 0.01; *** *p* < 0.001

To determine whether Purkinje cell loss is cause or consequence of Bergmann glia activation, we analysed the local correlation of Bergmann glia activation and Purkinje cell loss by GFAP/Calbindin co-staining (Fig. 5). We quantified the GFAP-positive area fraction in the molecular layer, as a measure of Bergmann glia activation, as well as Purkinje cell density in multiple fields and animals per experimental group (Fig. 5c and d). This analysis revealed that activation of Bergmann glia was arrested, but not reverted by transgene inactivation in line with the arrest of GFAP induction (see Fig. 3b and Additional file 1: Figure S4B), showing that Bergmann glia activation is irreversibly induced by transient IKK2 activation and thereby could trigger Purkinje cell loss independent of transgene repression (Fig. 5c and d). Supporting this model of Bergmann glia-driven Purkinje cell degeneration, we found degeneration exclusively in areas with activated Bergmann glia (Fig. 5a and c). Importantly, IKK2-CA repression did not prevent progression of Purkinje cell loss in areas with activated Bergmann glia (Fig. 5b-d). Virtually all areas with activated Bergmann glia displayed prominent Purkinje cell loss at 20 weeks although transgene expression was inactivated in the period of 12 to 20 weeks of age (Fig. 5b-d). This shows that Purkinje neurons are unable to survive for an extended time period in an environment of activated Bergmann glia.

Consistent with this model, in animals with continuous transgene expression some areas show Bergmann glia activation, but no or only moderate Purkinje cell loss (Fig. 5a and c-d), indicating that Bergmann glia in these areas have been activated only recently, and therefore the resulting delayed Purkinje cell loss is still in progress. Similarly, 2 weeks after transgene inactivation at 12 weeks of age, when inflammatory parameters are largely normalized (Additional file 1: Figure S4), most areas with Bergmann glia activation do not display prominent Purkinje cell degeneration yet (Fig. 5c and d). This indicates that Purkinje cell degeneration is a delayed process, which continues as a consequence of Bergmann glia dysfunction between 14 and 20 weeks (Fig. 5c and d).

To test the contribution of astrocyte-mediated microglia recruitment/activation on neurodegeneration, we performed a similar local correlation analysis of Purkinje cell degeneration and microglia activation by Iba1/Calbindin co-staining (Additional file 1: Figure S6A). This revealed that local microgliosis increases between 12 and 20 weeks if IKK2-CA is expressed whereas if the transgene is inactivated at 12 weeks, local microgliosis is almost fully reverted after 2 weeks (Dox12-14w, Additional file 1: Figure S6B and C), although mild residual microgliosis seems to remain even at the age of 20 weeks (Dox12-20w; Additional file 1: Figure S6B and C). Overall these findings confirm that microgliosis is almost fully reversible.

Remarkably, Purkinje cell degeneration is not significantly different between areas with and without microgliosis, even in the animals with active IKK2-CA and strong microgliosis (Additional file 1: Figure S6B and D), indicating that microglia activation, in contrast to Bergmann glia activation (Fig. 5), is not required for the progression of Purkinje cell degeneration.

**Fig. 6:**
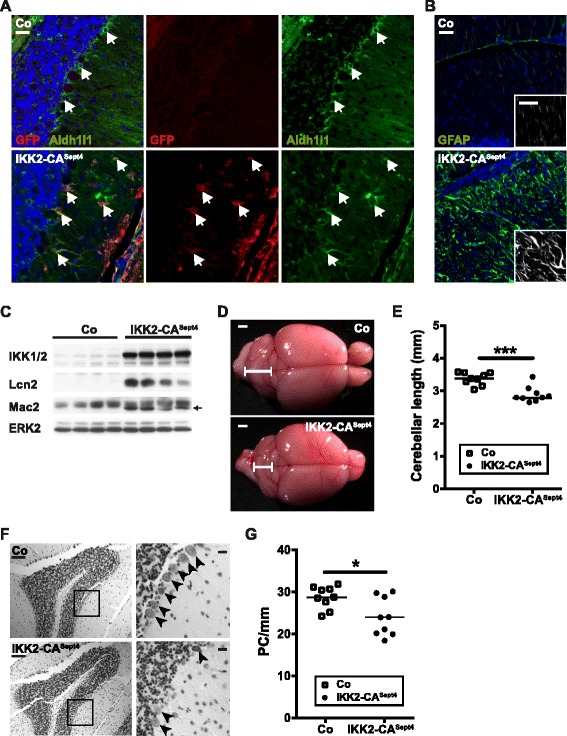
Bergmann glia specific IKK2-CA expression in the IKK2-CA^Sept4^ model recapitulates the cerebellar pathology of the GFAP.tTA/tetO.IKK2-CA mice. **a** Co-Immunostaining for the GFP reporter coexpressed with the IKK2-CA transgene and for Aldh1l1 as astrocyte marker shows co-localisation. Arrows indicate Bergmann glia cell bodies. Scale bar: 20 μm. **b** Immunostaining for GFAP shows intensly stained and unorganized Bergmann glia processes in the molecular layer indicating Bergmann glia activation in IKK2-CA^Sept4^ mice. Inlays: higher magnification of the GFAP channel in the molecular layer. Scale bars: 20 μm. **c** Immunoblotting for Mac2 indicates microgliosis, transgene expression (IKK 1/2 immunoreactivity) and induction of the NF-κB target Lcn2. **d** Sept4-Cre induced IKK2-CA expression results in macroscopic cerebellar atrophy at 28–29 weeks. Scale bar: 1 mm. **e** Quantification of maximal rostro-causal length of the cerebellum (single animals and mean); statistical analysis: 2-tailed unpaired *t*-test, *** *p* < 0.001. **f** Histological analysis of the simple lobule reveals loss of Purkinje cells in the IKK2-CA^Sept4^ model. Arrows: Purkinje cells. Scale bars: 100 μm (*left panels*); 20 μm (enlargements, *right panels*). **g** Quantification of Purkinje cell loss from Nissl stainings. Statistical analysis: 2-tailed unpaired *t*-test, * *p* < 0.05

Importantly, we found that the phenotype of GFAP.tTA/tetO.IKK2-CA mice is recapitulated also in a newly established, independent mouse model named Sept4-Cre/Rosa26-CAG-LSL-IKK2CA-IRESeGFP (short IKK2-CA^Sept4^). In this system, Sept4-Cre mediated recombination induces expression of IKK2-CA and a GFP reporter (Additional file 1: Figure S7A) specifically in Bergmann glia (Fig. 6a), but not in Purkinje cells (Additional file 1: Figure S7B), cerebellar granule neurons (Additional file 1: Figure S7C), microglia (Additional file 1: Figure S7D), and other astrocytes beside Bergmann glia, as shown for the cortex and corpus callosum (Additional file 1: Figure S7E). Similar to the doxycycline-driven pan-astrocyte expression of IKK2-CA, this results in astrogliosis-like alterations of Bergmann glia morphology with GFAP upregulation (Fig. 6a and b), and the upregulation of the NF-κB target gene Lcn2 (Fig. 6c). Also microgliosis is present in the cerebellum of the IKK2-CA^Sept4^ mice, shown by induction of the macrophage/microglia marker Mac2 (Fig. 6c) and immunostainings for Iba1 (Additional file 1: Figure S7D) Importantly, also IKK2-CA^Sept4^ animals display an atrophy of the cerebellum (Fig. 6d and e) and a loss of Purkinje cells (Fig. 6f and g). Although the phenotype develops later and is less pronounced than in the GFAP.tTA/tetO.IKK2-CA model, possibly due to reduced or delayed transgene expression, these findings overall confirm the critical role of IKK2 activation in Bergmann glia in the induction of Purkinje cell degeneration.

### Downregulation of astroglial glutamate transporters coincides with dark cell degeneration indicating excitotoxic neurodegeneration

**Fig. 7:**
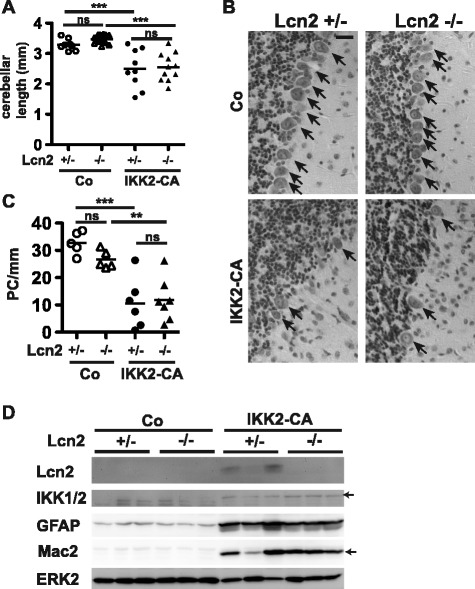
Lcn2 does not contribute to Purkinje cell loss, astrogliosis and microgliosis in IKK2-CA mice. **a** Cerebellar atrophy at 20 weeks of age. Measurement of maximal rostro-caudal length of the cerebellum (single animals and mean). Statistical analysis: 1-way-ANOVA, ns: not significant (*p* > 0.05); *** *p* < 0.001. **b**, **c** Loss of Purkinje cells (*arrows*) at 20 weeks. Nissl-stained images (**b**) and quantification in the simple lobule (**c**). Scale bars: 25 μm. Statistical analysis: 1-way-ANOVA, ns: not significant (*p* > 0.05); ** *p* < 0.01; *** *p* < 0.001. **d** Astrogliosis, microgliosis and IKK2-CA expression indicated by GFAP, Mac2 and IKK1/2. ERK2 is detected as loading control

We next asked how Bergmann glia activation might drive Purkinje cell loss. Beside the disruption of the structural support due to morphological alterations, Bergmann glia activation might also result in the production or impaired clearance of neurotoxic factors. One such putative neurotoxic factor is the NF-κB target gene Lcn2, which is secreted by astrocytes and is highly upregulated in the cerebellum of both the IKK2-CA and IKK2-CA^Sept4^ mice (Figs. 2i, j and 6c). Lcn2 was shown to be neurotoxic in vitro, and to critically contribute to the pathogenesis of CNS disorders in animal models [21–23]. In vitro studies also proposed Lcn2 as an important mediator of astrogliosis and microgliosis [24–27]. However, Lcn2 deficiency in IKK2-CA animals did affect neither cerebellar atrophy (Fig. 7a) nor Purkinje cell loss (Fig. 7b and c). Surprisingly, astrogliosis and microgliosis were also not altered by the lack of Lcn2, as depicted by immunoblotting for GFAP and Mac2 (Fig. 7d). This demonstrates that Lcn2 does not contribute to neuropathology in our model of inflammatory Purkinje cell degeneration.

**Fig. 8:**
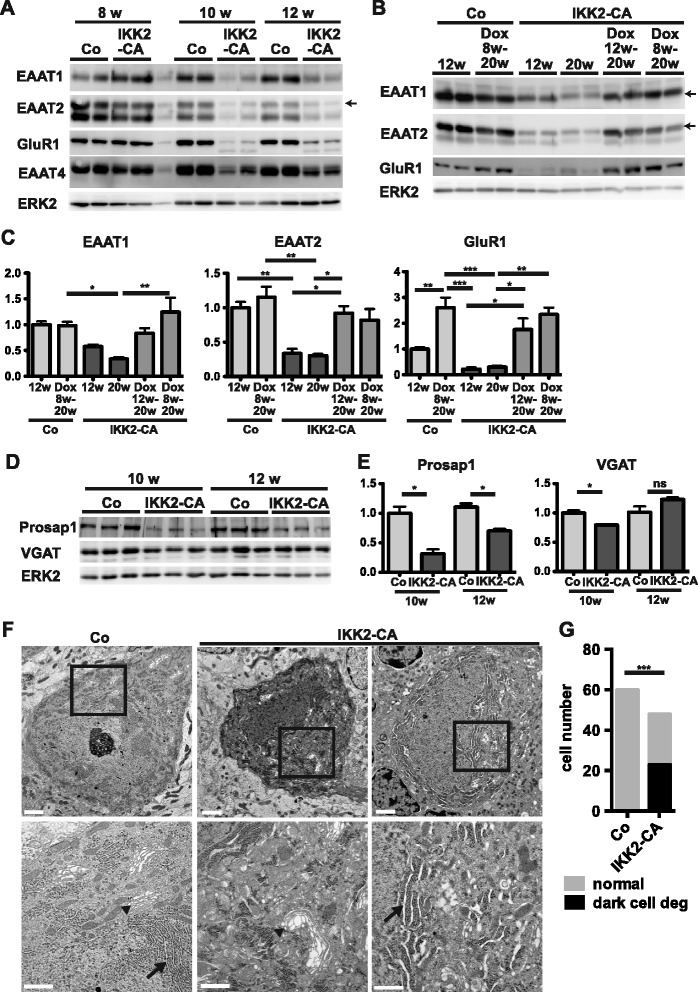
Reversible downregulation of glutamate transporters and GluR1 by IKK2-CA is associated with signs of synaptic degeneration and dark cell degeneration of Purkinje cells. **a** Downregulation of glial glutamate transporters EAAT1/2 and the AMPA-receptor subunit GluR1 by IKK2-CA. The Purkinje cell specific glutamate transporter EAAT4 is only moderately reduced. Re-probed membranes shown also in Fig. 2j (same ERK2 loading control). **b**, **c** Repression of IKK2-CA restores expression of EAAT1, EAAT2, and GluR1. Re-probed membranes shown also in Fig. 3b (same ERK2 loading control). Representative immunoblot (**b**) and quantification (**c**). Values normalized to ERK2, shown as mean +/- s.e.m. relative to Co 12w (*n* = 3–4). **d**, **e** Levels of the postsynaptic density protein Prosap1 are reduced, whereas there is no major change in the presynaptic protein VGAT. Immunoblot (**d**) with quantification (**e**). mean values +/- s.e.m. relative to Co 10w, normalized to ERK2 (loading control), statistical analysis: 2-tailed unpaired *t*-test (*n* = 3–4), ns: not significant; * *p* < 0.05. **f**-**g** Ultrastructural analysis shows darks cell degeneration of Purkinje cells in IKK2-CA animals (age 16 weeks) with darkened cytoplasm, irregular morphology and the dilatation of Golgi cisternae (*arrowheads*) and endoplasmatic reticulum (*arrows*). Scale bars: 2 μm (*upper panels*), 1 μm (*lower panels*). **g** Quantification of *n* = 60 Purkinje cells of controls and *n* = 48 Purkinje cells of IKK2-CA animals (pooled of each 3 animals); statistical analysis: Fisher’s exact test, *p* < 0.0001

Another well-known factor implicated in neurodegeneration is the neurotransmitter glutamate. Several studies attributed Purkinje cell loss to excitotoxicity as consequence of downregulation of the glutamate transporters EAAT1 and EAAT2 in Bergmann glia, resulting in impaired clearance of extracellular glutamate [17–19]. Notably, in GFAP/IKK2-CA animals we found a pronounced downregulation of EAAT1 and EAAT2 already at 10 weeks (Fig. 8a) correlating with transgene expression (Fig. 2j) and in line with a potential pathogenic role in ataxia and Purkinje cell loss. Accordingly, we observed downregulation of EAAT1 and EAAT2 also in the IKK2-CA^Sept4^ model (Additional file 1: Figure S8A).

In addition, we identified a pronounced reduction of the AMPA receptor subunit GluR1 in GFAP/IKK2-CA mice, indicating additional alterations in glutamatergic signalling (Fig. 8a). Interestingly, downregulation of EAAT1, EAAT2 and GluR1 is fully reversible upon IKK2-CA transgene inactivation at 20 weeks of age (Fig. 8b and c). EAAT2 levels are already normalized after 2 weeks of doxycycline application (Additional file 1: Figure S8B). This finding indicates that continuous glutamate transporter downregulation of EAAT1 and EAAT2 is not required for the progression of Purkinje cell loss, as transgene repression from 12 weeks of age can reconstitute EAAT1/2 and GluR1 expression, but cannot prevent Purkinje cell loss. However, transgene inactivation at 8 weeks, a time point at which no obvious changes in EAAT1/2 expression are seen (see Fig. 3) prevented Purkinje cell degeneration. Remarkably, downregulation of EAAT2 is not seen throughout the brain (Additional file 1: Figure S8C and D) but is rather restricted to the cerebellum and to a lesser extent also observed in the medulla (Additional file 1: Figure S8E and F). This may argue for a cell-type specific effect of IKK2 mediated EAAT1/2 regulation in a subset of astrocytes, most notably in Bergmann glia.

In contrast to the glial glutamate transporters, expression of EAAT4, a glutamate transporter expressed specifically by Purkinje cells [28] is only slightly reduced at the age of 10 and 12 weeks (Fig. 8a), excluding a major loss of Purkinje cells as well as compensatory induction of EAAT4 at this early stages. Furthermore, at a later stage (age 36 weeks), we could not detect compensatory induction of the neuronal glutamate transporters EAAT3 and EAAT4 in the cerebellum (Additional file 1: Figure S8G). Instead, EAAT4 expression was prominently decreased, well correlating with the massive loss of Purkinje neurons at this time point. In addition, reduction in EAAT3 levels also suggested loss of other neuronal cells in the cerebellum in addition to Purkinje neurons (Additional file 1: Figure S8G).

EAAT1 and EAAT2 have been described as genes that can be directly suppressed by NF-κB in specific conditions [29, 30]. However, unexpectedly, EAAT1/2 mRNA levels are not altered in IKK2-CA animals (Additional file 1: Figure S8H), demonstrating that downregulation of these transporters is not caused by direct IKK2/NF-κB mediated transcriptional repression, but is rather due to a posttranscriptional mechanism, regulating EAAT1/2 translation or protein stability. Interestingly, in silico analysis of EAAT1/2 mRNA sequences using the online tool TargetScanMouse 7.1 identified several putative binding sites for the NF-κB regulated miRNA miR-146a, which is highly upregulated in the IKK2-CA animals (Additional file 1: Figure S8I). Therefore, miRNA mediated translational inhibition via NF-κB regulated miR-146a represents a potential mechanism for downregulation of EAAT1/2 protein levels in IKK2-CA mice, an issue, which needs detailed analysis in the future.

Supporting the hypothesis that excitotoxicity is involved in the initiation of neurodegeneration, we found reduced expression of the postsynaptic density protein Prosap1/Shank2 already at the age of 10 weeks in the cerebellum, whereas VGAT expression as a marker of the axonal/presynaptic compartment of the GABAergic Purkinje neurons was only marginally altered (Fig. 8d and e). As excitotoxicity is a consequence of local hyperexcitation of individual synapses, the postsynaptic/dendritic compartment might display an early damage before other parts of Purkinje cells get affected. Finally, we found ultrastructural alterations described as dark cell degeneration, which have been shown to occur in Purkinje cells dying by excitotoxicity. Dark cell degeneration is characterized by a darkened cytoplasm, dilated Golgi cisternae, and a dilated endoplasmatic reticulum [18, 31]. At 16 weeks, we could detect such alterations in 48% of analysed Purkinje cells in IKK2-CA animals but never in controls (Fig. 8f and g).

## Discussion

In the present study, we demonstrate that genetic activation of IKK2 in astrocytes and especially in Bergmann glia results in chronic neuroinflammation and subsequent cerebellar ataxia with a rather selective degeneration of Purkinje neurons in adult mice. Degeneration is caused via a non-cell-autonomous mechanism disrupting the essential local Bergmann glia Purkinje cell interaction. Importantly, only a restricted time period of neuroinflammation is sufficient to initiate subsequent degeneration of Purkinje neurons. In addition, we show that inflammation-associated astrogliosis induced by IKK2 activation results in a strong downregulation of the glial glutamate transporters EAAT1 and EAAT2 in the cerebellum, most likely leading to a local imbalance of glutamate homeostasis and promoting excitotoxicity.

Chronic neuroinflammation is increasingly recognized as a pathogenic mechanism in neurological disorders, especially in neurodegeneration [2]. Remarkably, distinct neuronal populations show different degrees of vulnerability to neurodegeneration, a phenomenon that is not well understood. This selective vulnerability is usually attributed to cell intrinsic properties of the affected neurons, although interactions with glial cells and local inflammatory conditions also might play a role [32].

Interestingly, depending on the experimental setup, inflammation alone can be sufficient to induce degeneration of vulnerable neurons. In a model of LPS-induced chronic neuroinflammation, degeneration of dopaminergic neurons was detected [33], whereas in the IKK2-CA model the survival of dopaminergic neurons is not affected, despite global chronic neuroinflammation [15]. Instead we found a selective degeneration of Purkinje neurons, which is a pathological hallmark of inflammatory cerebellar ataxias. In these neurodegenerative disorders, a variety of strong neuroinflammatory insults, e.g. autoimmune disorders like paraneoplastic syndromes, converge on a selective degeneration of Purkinje cells [34–36].

Our study supports a comprehensive model how diverse inflammatory insults could cause cerebellar ataxia: Disease-mediated elevation of danger signals like TLR ligands and inflammatory cytokines in the cerebellum is able to activate IKK2 signalling in astrocytes [2, 3], which as a consequence triggers astrogliosis-like activation of Bergmann glia and subsequent Purkinje cell loss. In favour of this hypothesis, genetic inactivation of Myd88, a critical signal transduction component in TLR signalling and important for downstream IKK/NF-κB activation, alters neuroinflammation and attenuates Purkinje cell loss in a spinocerebellar ataxia type 6 mouse model [37]. Several studies indicate that the pathology of inflammatory cerebellar ataxia is very similar to the one observed in the IKK2-CA model, importantly also showing prominent astrogliosis including activation of Bergmann glia [38–42].

With the detailed analysis of the local correlation of Purkinje cell death and Bergmann glia activation together with our rescue approach and Bergmann glia specific IKK2 activation in IKK2-CA^Sept4^ mice we provide strong evidence that such astrogliosis-like activation of Bergmann glia is sufficient to trigger Purkinje cell degeneration and explains the selective vulnerability of Purkinje cells to inflammatory insults. In contrast, our experiments do not indicate a similar crucial role of microglia activation in Purkinje cell degeneration. Overall these findings support the idea that IKK2-driven Bergmann glia activation is able to cause Purkinje cell loss in inflammatory cerebellar ataxias and suggest that the IKK2-CA model can serve as a useful tool to study these disorders. The detailed analysis of the IKK2-CA^Sept4^ model also revealed that the Sept4-Cre driver line, which was generated by the GENSAT project [43], but was not thoroughly characterized, can be used to induce Cre-mediated recombination selectively in Bergmann glia, thus providing a tool to study gene function specifically in Bergmann glia without targeting other astrocytes.

Most known functions of IKK2 in the regulation of inflammatory processes are linked to its function in NF-κB signalling. In line with this, we found IKK2-CA mediated NF-κB activation in Bergmann glia measured by nuclear localisation of p65. In addition, our gene expression analyses revealed the upregulation of several proinflammatory NF-κB target genes in the cerebellum upon IKK2-CA expression and their immediate downregulation upon doxycycline-mediated transgene inactivation. However, it remains open so far and needs to be addressed in future studies, whether NF-κB signalling or NF-κB-independent IKK2 phosphorylation substrates like IRS1, Foxo3A, SNAP23 or TSC1 [44] are mediating Bergmann glia activation and subsequent Purkinje cell degeneration.

The results obtained with the conditional inactivation of the transgene also revealed a surprising aspect, namely that astrogliosis-like Bergmann glia activation is irreversible and determines an early “point of no return” resulting in inevitable Purkinje cell loss. The phenomenon of such an early “point of no return” preceding actual neuron loss is observed in different neurodegenerative disorders, which complicates therapeutic approaches [45]. Whereas this phenomenon is usually attributed to cell intrinsic mechanisms of neurons, e.g. synaptic dysfunction, our data show that it can also depend on an irreversible astrocyte defect. Overall our data implicate that early intervention is crucial for the development of therapeutic strategies for treatment of inflammatory cerebellar ataxias.

The nature of the specific support functions of Bergmann glia required for Purkinje cell survival requires further investigation. Beside loss of structural support, activation of Bergmann glia might induce the production of toxic substances or impair the clearance of such substances. In contrast to its pathogenic role in the parkinsonian dopaminergic system [46] and in other model systems [21–23], we could exclude any contribution of the secreted neurotoxic factor Lcn2 to Purkinje cell degeneration.

In fact, our data indicate that impaired clearance of glutamate via the glutamate transporters EAAT1 and EAAT2 by Bergmann glia could be responsible for Purkinje cell degeneration. Similar to our findings, other studies [17–19] found a downregulation of these transporters in astrocyte-mediated Purkinje cell degeneration and indirect evidence for altered glutamate homeostasis. A recent study shows hyperexcitability of Purkinje cells in response to IL-1β mediated downregulation of EAAT1 and Bergmann glia activation in EAE [47]. As excitotoxicity is a consequence of neuronal hyperexcitation, these findings support the hypothesis that inflammation-associated impairment of glutamate uptake by Bergmann glia causes Purkinje cell excitotoxicity.

Our finding that restoration of EAAT1/2 expression after transgene inactivation at 12 weeks of age did not prevent progression of Purkinje cell degeneration highlight the notion that persistent excitotoxicity is not necessary to induce Purkinje cell death. In contrast, a limited time period of deregulated glutamate homeostasis is sufficient to cause this irreversible Purkinje cell damage. Furthermore, glutamate uptake could additionally be compromised by dislocation of EAAT1/2 from Purkinje cell synapses due to an activation-related retraction of Bergmann glia processes.

The reversible IKK2-dependent repression of EAAT1 and EAAT2 in vivo is also of broader relevance, as neuroinflammation and excitotoxicity are supposed to be involved in the pathogenesis of various neurodegenerative diseases, but the molecular link between both processes is not well understood [48]. Interestingly, several IKK/NF-κB activating cytokines can reduce glutamate transporter expression and glutamate uptake in astrocytes [48], although cell culture studies produced conflicting results on the role of NF-κB in EAAT1/2 regulation [29, 30, 49]. Our data now clearly indicate that IKK2 in cerebellar astrocytes acts as negative regulator of EAAT1 and EAAT2 expression in vivo, remarkably not by direct NF-κB-mediated transcriptional repression, but via a posttranscriptional mechanism, reducing EAAT1/2 protein levels without affecting mRNA expression. While initial results show an induction of miR-146a, a NF-κB inducible miRNA, which is predicted to target both EAAT1 and EAAT2 mRNAs and could thereby inhibit mRNA translation, other possible mechanisms need to be explored in future studies, including the possibility of a NF-κB independent regulation of EAAT1/2 protein stability by IKK2.

Overall, these findings revealed an unexpected aspect of the regulation of glutamate homeostasis by the IKK2 system, suggesting a novel link between neuroinflammation and deregulation of glutamate homeostasis in CNS disorders.

## Conclusions

Our results implicate that inflammation-mediated dysfunction of a specific astrocyte population is sufficient to determine the selective vulnerability of neurons to degeneration, an aspect that so far receives only limited attention. Importantly, this novel non-cell-autonomous mechanism significantly improves the understanding how diverse insults by inducing IKK2 activation and NF-κB-mediated inflammation could cause inflammation/autoimmune-associated cerebellar ataxias.

## Methods

### Transgenic mice

GFAP.tTA/tetO.IKK2-CA or GFAP.tTA/tetO.IKK2-DN double transgenic mice named IKK2-CA or IKK2-DN were described previously [14]. For IKK2-CA repression doxycycline (0,5 g/l) was administered in the drinking water of all mice from conception to 4 weeks of age and as indicated in Fig. 3a and Additional file 1: Figure S4A. Both male and female mice were included (no difference in phenotype, data not shown), and similarly doxycycline treated tetO.IKK2-CA or GFAP.tTA single transgenic mice and wildtype littermates were used as controls (single transgenic mice were indistinguishable from wildtype, data not shown). To analyse the contribution of Lcn2, the Lcn2 null allele *Lcn2* ^*tm1Aade*^ [50] was bred into the GFAP/IKK2-CA line. Sept4-Cre mice (Tg(Sept4-cre)OX54Gsat/Mmucd, MGI ID: MGI:5086169) were generated by the GENSAT Project at Rockefeller University [43] and obtained by the ‘Mutant Mouse Resource Research Centers’ (Gensat, RRID:MMRRC_036147-UCD). Sept4-Cre mice are described to give rise to Cre-mediated recombination in cerebellar glia cells (subtype, Bergmann glia; http://www.gensat.org/), which was validated by co-staining analyses in this study (Fig. 6 and Additional file 1: Figure S7). To generate Rosa26-CAG-LSL-IKK2CA-IRESeGFP mice the targeting vector was placed into the Rosa26 locus (Additional file 1: Figure S7A) via electroporation of C57BL/6-derived embryonic stem (ES) cells. Correctly targeted ES cells were selected and chimeric animals were bred to C57BL/6 mice to generate mutant mice. Sept4-Cre mice were crossed to Rosa26-CAG-LSL-IKK2CA-IRESeGFP mice (Additional file 1: Figure S7A) to generate double transgenic Sept4-Cre/Rosa26-CAG-LSL-IKK2CA-IRESeGFP mice termed IKK2-CA^Sept4^ in order to express IKK2-CA and eGFP in Bergmann glia. All mice were of a pure C57BL/6 genetic background. Both male and female mice were included and single transgenic mice and wildtype littermates were used as controls.

### Rotarod and beam-walking test

Fast movement coordination was analysed with the ENV-575 M rotarod (Med Associates Inc.). After 1 min at 4 rpm for adjustment, the cylinder accelerated within 5 min to 40 rpm. The latency to fall was recorded. To analyse motor learning, each animal was subjected to the task 3 times per day for 4 consecutive days.

In the beam-walking test, the mice had to traverse a narrow beam to escape from a small, elevated platform to a closed dark box, with subtle encouragement by the experimenter. Beginning from the second trial for each trial the crossing time was recorded. For the first experiment (Fig. 1) a protocol with 4 training trials per day for 3 days with a 12 mm square beam (length 80 cm) was used. On the two following days, probe trials with different beam sizes were done in duplicate. Other experiments were performed with 4 consecutive trials on 1 day with a 12 mm square beam.

### High-resolution MRI

Experiments were carried out under isoflurane anesthesia (5% for induction, 1.5% for maintanence, mixed with air). All Data were acquired on a dedicated small animal MRI system (BioSpec 117/16 USR, Bruker Biospin, Ettlingen, Germany) applying a two-element cryogenically cooled transmit/receive surface coil. The animals were positioned in prone position with the head fixed to a purpose-built head holder and nose cone. Body temperature was maintained at 37 °C using a water heated animal bed. T2*-weighted images were acquired applying a FLASH sequence with acquisition parameters as: TR/TE = 190/5 ms, flip angle a = 17.5°, slice thickness s = 0.5 mm, in-plane resolution Dr = 65 x 65 μm^2^. For coverage of the entire cerebellum 18 slices without any interslice gap were acquired in a total measurement time TACQ = 10 min.

### Protein isolation and immunoblotting

For tissue protein extracts brain regions were snap-frozen in liquid nitrogen, grinded while frozen and lysed in RIPA buffer (50 mM Tris-HCl, 150 mM NaCl, 1% Triton X-100, 0.5% sodium deoxycholate, 0.1% SDS, pH 7.4) supplemented with protease inhibitors (1 mM PMSF and Roche “complete mini” -tablets). Non-lysed debris was removed by centrifugation (25 min, 17000 g).

Equal amounts of protein (usually 20–50 μg) were seperated by SDS-PAGE under reduced-denaturing conditions. For an improved dissociation of glutamate transporter oligomers, samples were usually denatured with a twofold concentrated urea supplemented Laemmli loading buffer (200 mM Tris-HCl, 15% glycerol, 4% SDS, 6 M Urea, 5% β-mercaptoethanol). Proteins were transferred to PVDF membranes and blocked for 1 h with 5% dry milk in TBS buffer. Primary antibodies were incubated in the blocking solution overnight at 4 °C or for 2 h at room temperature, secondary antibodies for 1 h at room temperature. Luminescence signals were detected with the “Intelligent Dark Box” (Fuji). The membranes were usually reprobed several times with different antibodies, including anti-ERK2 as loading control.

### RNA extraction, cDNA synthesis and qPCR

RNA was prepared with the Peqlab Trifast kit and cDNA synthesized with the Roche Transcriptor High fidelitiy cDNA synthesis kit with oligo-dT primers. Quantitative Realtime-PCR (qPCR) assays designed with the Roche “Universal Probe Library” system were run on the Roche Lightcycler 480. Values were normalized to HPRT as housekeeping gene. Intron spanning primers for qPCR assays were designed with the online “Roche Universal ProbeLibrary Assay Design Center” and used with the respective fluorescent UPL probe in standard assay conditions for the Roche Universal Probe qPCR system. Sequences are as follows:

HPRT1 (5’-gga gcg gta gca cct cct-3’, 5’-ctg gtt cat cat cgc taa tca c-3’, UPL #69), MCP-1(5’-cat cca cgt gtt ggc tca-3’, 5’-gat cat ctt gct ggt gaa tga gt-3’, UPL #62), Rantes (5’-tgcagaggactctgagacagc-3’, 5’-gagtggtgtccgagccata-3’, UPL #110), IL-1β (5’-tgt aat gaa aga cgg cac acc-3’, 5’-tct tct ttg ggt att gct tgg-3’, UPL #78), IL-6 (5’-gct acc aaa ctg gat ata atc agg a-3’, 5’-cca ggt agc tat ggt act cca gaa-3’, UPL # 6), ICAM-1 (5’-ccc acg cta cct ctg ctc-3’, 5’-gat gga tac ctg agc atc acc-3’, UPL #81), Madcam-1 (5’-gggcaggtgaccaatctgta-3’, 5’-ataggacgacggtggagga-3’, UPL #72), Lcn2 (5’-ccatctatgagctacaagagaacaat-3’, 5’-tctgatccagtagcgacagc-3’, UPL #58), C3 (5’-accttacctcggcaagtttct-3’, 5’-ttgtagagctgctggtcagg-3’, UPL #76), C4b (5’-tctcacaaacccctcgacat-3’, 5’-agcatcctggaacacctgaa-3’, UPL #10), CD74 (5’-gccctagagagccagaaagg-3’, 5’-tggtacaggaagtaagcagtgg-3’, UPL #21), H2-Aa (5’-tggaggtgaagacgacattg-3’, 5’-ctcatcaccatcaaattcaaatg-3’, UPL #80), TNF (5’-tgcctatgtctc- agcctcttc-3’, 5’-gaggccatttgggaacttct-3’, UPL #49).

For the analysis of miR-146a expression, total RNA was prepared with the Qiagen “miRNeasy” kit and qPCR performed with the Qiagen Quantitect primer assays for miR-146a and RNU-2 as housekeeping gene.

### Histology and immunofluorescence staining

Brains for histology were fixed by immersion with 4% PFA (3-5 h or overnight at 4 °C), dehydrated and embedded in paraffin. Coronal sections were prepared with a thickness of 7 μm. After rehydration, heat mediated antigen retrieval was performed with sodium citrate (10 mM, pH 6, 0.05% Tween 20) or Tris-EDTA (10 mM Tris, 1 mM EDTA, pH 9, 0.05% Tween 20). For full permeabilization, sections were incubated with 0.5% Triton X-100 for 30 min. Sections were then washed with PBS and blocked with 5% BSA in PBS for 1 h. Incubation with the primary antibodies (in 5% BSA) was performed overnight at 4 °C, secondary antibodies (in 5% BSA) were applied for 1 h at room temperature with 100–250 ng/ml DAPI for nuclear counterstaining.

Stainings for CD45, CD4, CD8, CD11b and Mac-2 were performed with 10 μm kryosections from natively frozen brains that were fixed with cold methanol (- 20 °C). Blocking and staining was performed as described above. The directly PE-labeled rat anti-CD11b antibody was applied for 2 h after careful washing after the secondary antibody, or directly after blocking, when only directly labeled antibodies were used.

Fluorescence images were acquired with the Zeiss Axiovert 200 M microscope with filters for DAPI, FITC/Alexa Fluor 488, DsRed/PE and TexasRed/Alexa Fluor 568/594, with 5/10/20x objectives and the Zeiss Axiovision software, except for Fig. 6a and b, Additional file 1: Figures S3F and S7 where images were aquired with the Keyence BZ-9000 (BIOREVO) microscope and the BZ-II Viewer software. For each channel, exposure times were separately adjusted and kept for the complete session. Adjustment of contrast and brightness was performed for each channel separately, but in all compared pictures equally.

### Quantification of the correlation of Bergmann glia or microglia activation and Purkinje cell loss

For quantification of the correlation of Bergmann glia or microglia activation and Purkinje cell loss, images of the cerebellar molecular layer and the adjacent Purkinje cell layer in GFAP/Calbindin or Iba1/Calbindin co-stainings were acquired in one session with a 20x objective. For quantification of Bergmann glia activation, a region of interest encompassing the major area of the molecular layer was defined, where the local GFAP positive area fraction (area above a manually set threshold vs. total area) was measured by ImageJ. For quantification of microglia activation, a region of interest including the major area of the molecular layer, Purkinje cell layer and granule cell layer was defined, where the local Iba1 positive area fraction (area above a manually set threshold vs. total area) was quantified. Then for the specific image the number of Purkinje cells was counted and the length of the Purkinje cell layer measured to calculate the local Purkinje cell density.

### Antibodies for immunoblotting and immunofluorescence

The following antibodies were used for immunoblotting: Mouse anti-calbindin (Sigma), rabbit anti-EAAT2 (Cell Signaling Technology), mouse anti-NeuN (Millipore), Rabbit anti-GluR1 (Millipore), rabbit anti-EAAT4 (Abcam) and goat anti-Lcn2 (R&D Systems). Goat anti-IKK2 (human specific, detects only the transgene), rabbit anti-IKK1/2, rabbit anti-ERK2, rabbit anti-EAAT1, rabbit anti-EAAT3, rabbit anti-galectin 3 (Mac-2), mouse anti-GFAP and all corresponding HRP coupled secondary antibodies were obtained from Santa Cruz Biotechnologies.

Rabbit anti-Prosap1 was kindly provided by Prof. Tobias Böckers (Ulm, Germany).

Goat anti-IKK2, mouse anti-GFAP, mouse anti-NeuN and mouse anti-calbindin were also used for immunofluorescence. Additionally, following antibodies were used for immunofluorescence: mouse anti-Aldh1l1 (Abcam), rabbit anti-RelA (Santa Cruz Biotechnologies), PE-labeled rat anti-CD11b (eBioscience), and rat anti-CD45, rat anti-CD8, rat anti CD4 (all from BD Biosciences), rabbit anti-Iba1 (Wako) and chicken anti-GFP (Abcam). Corresponding Alexa Fluor-conjugated secondary antibodies were obtained from Molecular Probes (Life Technologies).

### Electron microscopy

The protocol for the TEM studies on Purkinje cells was adapted from the work of Custer et al. [18]. Mice were anesthetized and transcardially perfused with PBS followed by 4% PFA/0.5% glutaraldehyde and then by 2% PFA/3% glutaraldehyde in PBS. After excision the whole brain was postfixed for at least overnight by immersion in 2% PFA/3% glutaraldehyde in PBS. The cerebella were cut with a vibratome into 0.5 mm thick coronal sections. Of these sections, pieces of the cerebellum containing the simple lobule were dissected. Tissue pieces were stained with osmium tetroxide and uranyl acetate and epoxy embedded. Semi-thin sections were prepared and stained with toluidine blue to select Purkinje cell containing areas for ultrathin sections. Ultrathin sections were stained with lead citrate and images of individual Purkinje cells (about 20 per animal if possible) were acquired with the JEM-1400 (JEOL, Tokyo, Japan) at a lower magnification to show the whole cell body and a higher magnification to visualize ER and Golgi structures. Purkinje cells were graded by three criteria, namely darkened cytoplasm, ER/Golgi swelling and irregular cell shape, on a scale of each 0 (normal) to 2 (prominently altered). Cells that had the score 2 in at least 2 criteria were regarded as degenerating.

### Image analysis and statistical analysis

Immunoblot densitometry and quantifications from microscopy images were performed with ImageJ. Statistical analysis was performed with Graphpad Prism as indicated in the specific figures.

## Additional file

Additional file 1: Figure S1. Additional phenotypic characterization of IKK2-CA and IKK2-DN mice. **Figure S2.** Additional immune cell markers and proinflammatory genes characterizing IKK2-CA-induced cerebellar neuroinflammation. **Figure S3.** Neuroinflammation is also found in other brain regions, but neurodegeneration is restricted to the cerebellum. **Figure S4.** Expression kinetics of inflammatory mediators upon transgene inactivation in IKK2-CA mice. **Figure S5.** The IKK2-CA transgene is not expressed in Purkinje cells or microglia. **Figure S6.** Local microglia activation is not sufficient to drive Purkinje cell degeneration. **Figure S7.** Characterisation of Bergmann glia specific expression of the IKK2-CA-IRES-GFP transgene in the IKK2-CA^Sept4^ model. **Figure S8.** Characterization of glutamate transporter expression in response to astroglial IKK2 activation. (PDF 20468 kb)

## Abbreviations

Aldh1l1: Aldehyde dehydrogenase 1 family member L1
EAAT: Excitatory amino acid transporter
GABA: Gamma-aminobutyric acid
GFAP: Glial Fibrillary Acidic Protein
GFP: Green Fluorescent Protein
GluR1: AMPA receptor subunit
Iba1: Ionized calcium binding adapter molecule 1
IKK: I-kappaB-kinase
Mac2: Mac2 antigen (Galectin 3)
NeuN: NeuN antigen (Neuronal nuclei)
NF-κB: Nuclear factor-kappa B
TLR: Toll-like receptor
VGAT: Vesicular GABA transporter
