# Supplementary material for: Transient IKK2 activation in astrocytes initiates selective non-cell-autonomous neurodegeneration
Source: Mol Neurodegener. 2017 Feb 13;12:16. doi: 10.1186/s13024-017-0157-0 (PMC5307695; doi:10.1186/s13024-017-0157-0)
Supplement: Additional file 1: Figure S1. — Additional phenotypic characterization of IKK2-CA and IKK2-DN mice. Figure S2. Additional immune cell markers and proinflammatory genes characterizing IKK2-CA-induced cerebellar neuroinflammation. Figure S3. Neuroinflammation is also found in other brain regions, but neurodegeneration is restricted to the cerebellum. Figure S4. Expression kinetics of inflammatory mediators upon transgene inactivation in IKK2-CA mice. Figure S5. The IKK2-CA transgene is not expressed in Purkinje cells or microglia. Figure S6. Local microglia activation is not sufficient to drive Purkinje cell degeneration. Figure S7. Characterisation of Bergmann glia specific expression of the IKK2-CA-IRES-GFP transgene in the IKK2-CASept4 model. Figure S8. Characterization of glutamate transporter expression in response to astroglial IKK2 activation. (PDF 20468 kb) [file 13024_2017_157_MOESM1_ESM.pdf]

Additional file

Transient IKK2 activation in astrocytes initiates selective non-cell-autonomous neurodegeneration

Michael Lattke, Stephanie N. Reichel, Alexander Magnutzki, Alireza Abaei, Volker Rasche, Paul Walther, Dinis P. Calado Boris Ferger, Thomas Wirth and Bernd Baumann

Contains:

Figures S1 – S8.

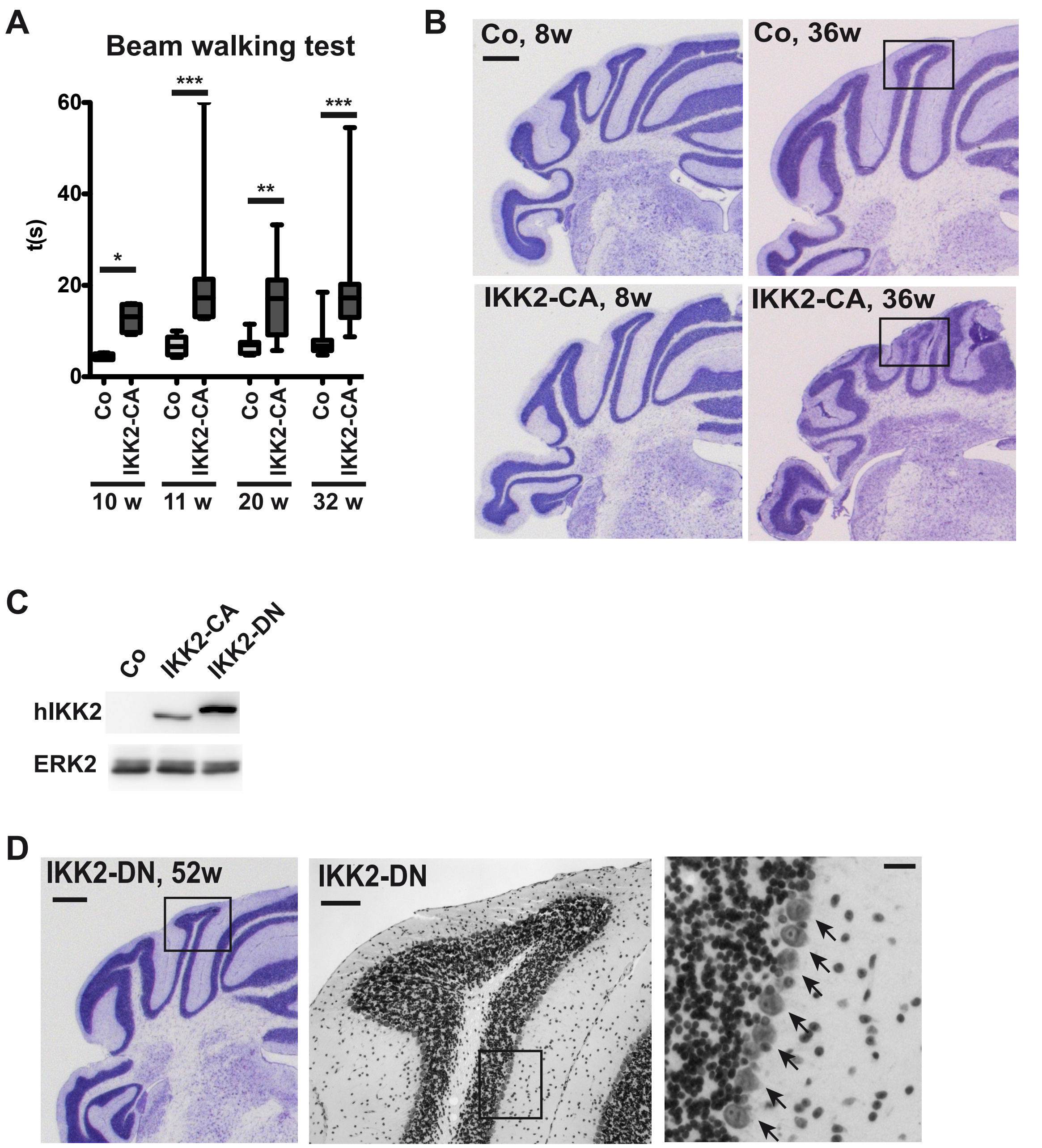
A
Beam walking test
t(s)
60
40
20
0
*
***
**
***
Co
IKK2-CA
10 w
11 w
20 w
32 w
B
Co, 8w
Co, 36w
IKK2-CA, 8w
IKK2-CA, 36w
C
Co
IKK2-CA
IKK2-DN
hIKK2
ERK2
D
IKK2-DN, 52w
IKK2-DN

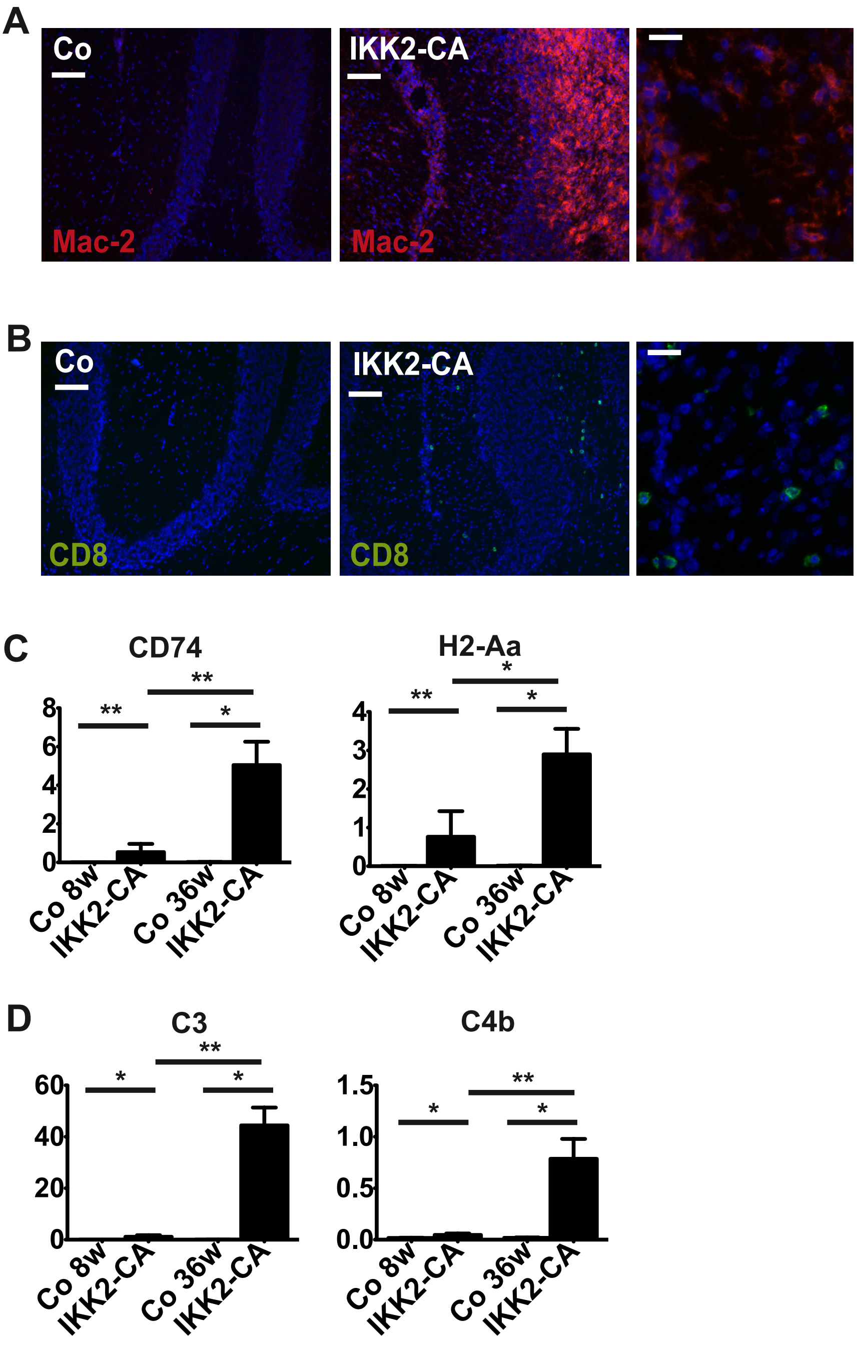
A
Co
Mac-2
IKK2-CA
Mac-2
B
Co
CD8
IKK2-CA
CD8
C
CD74
8
6
4
2
0
**
**
*
Co 8w
IKK2-CA
Co 36w
IKK2-CA
H2-Aa
4
3
2
1
0
**
*
*
Co 8w
IKK2-CA
Co 36w
IKK2-CA
D
C3
60
40
20
0
*
**
*
Co 8w
IKK2-CA
Co 36w
IKK2-CA
C4b
1.5
1.0
0.5
0.0
*
**
*
Co 8w
IKK2-CA
Co 36w
IKK2-CA

Lattke et al., Figure S3

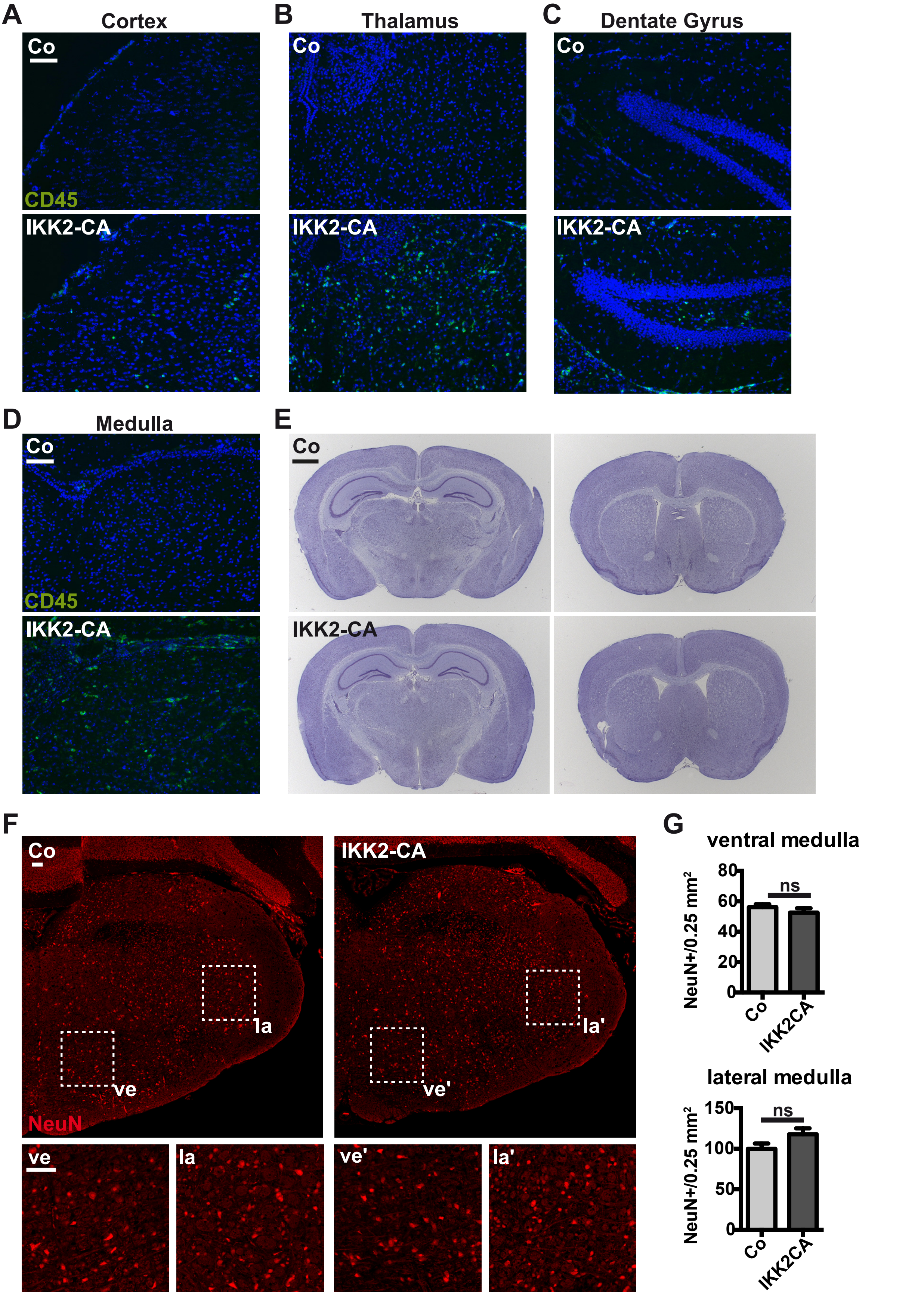

Lattke et al., Figure S4

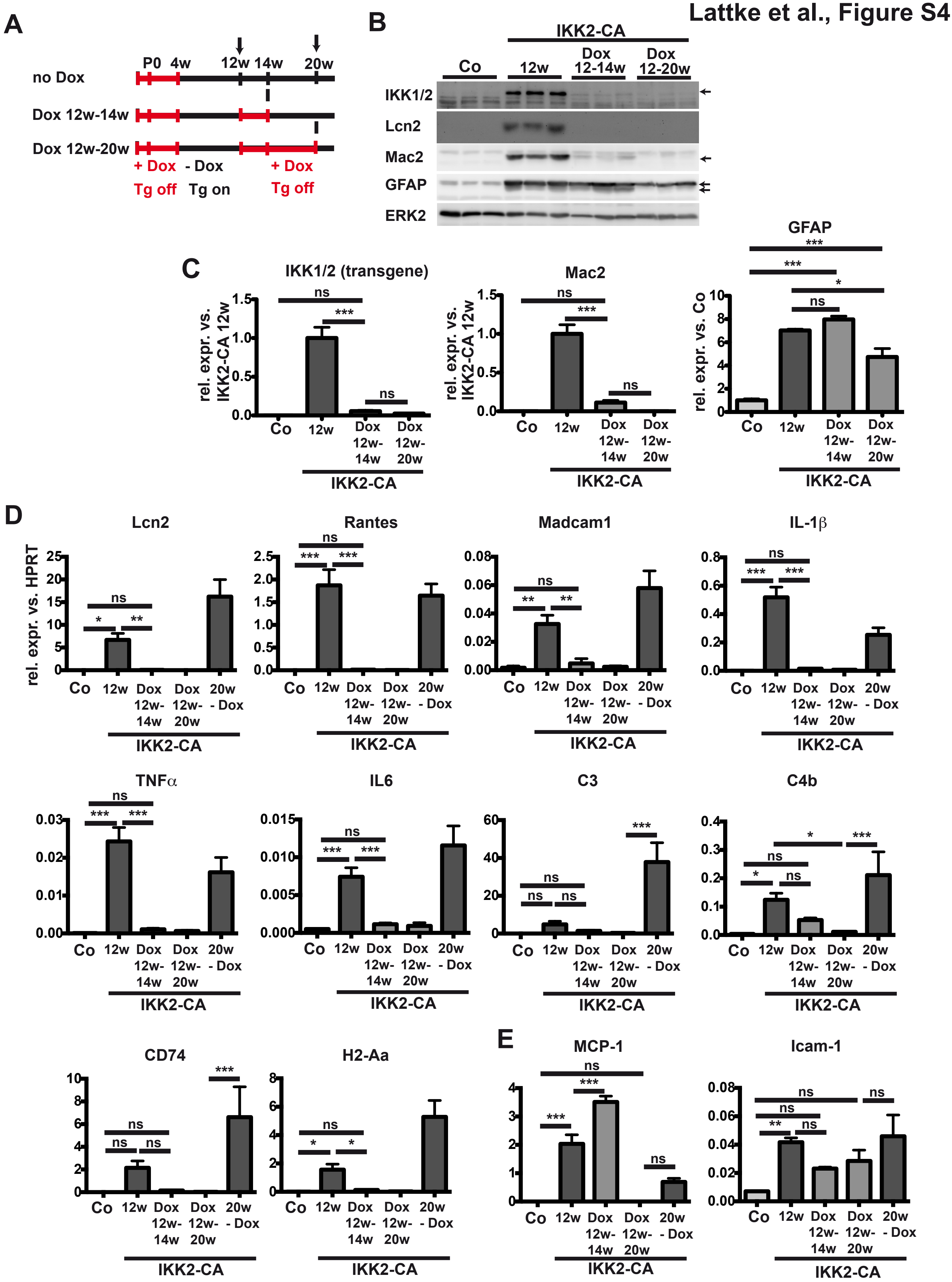

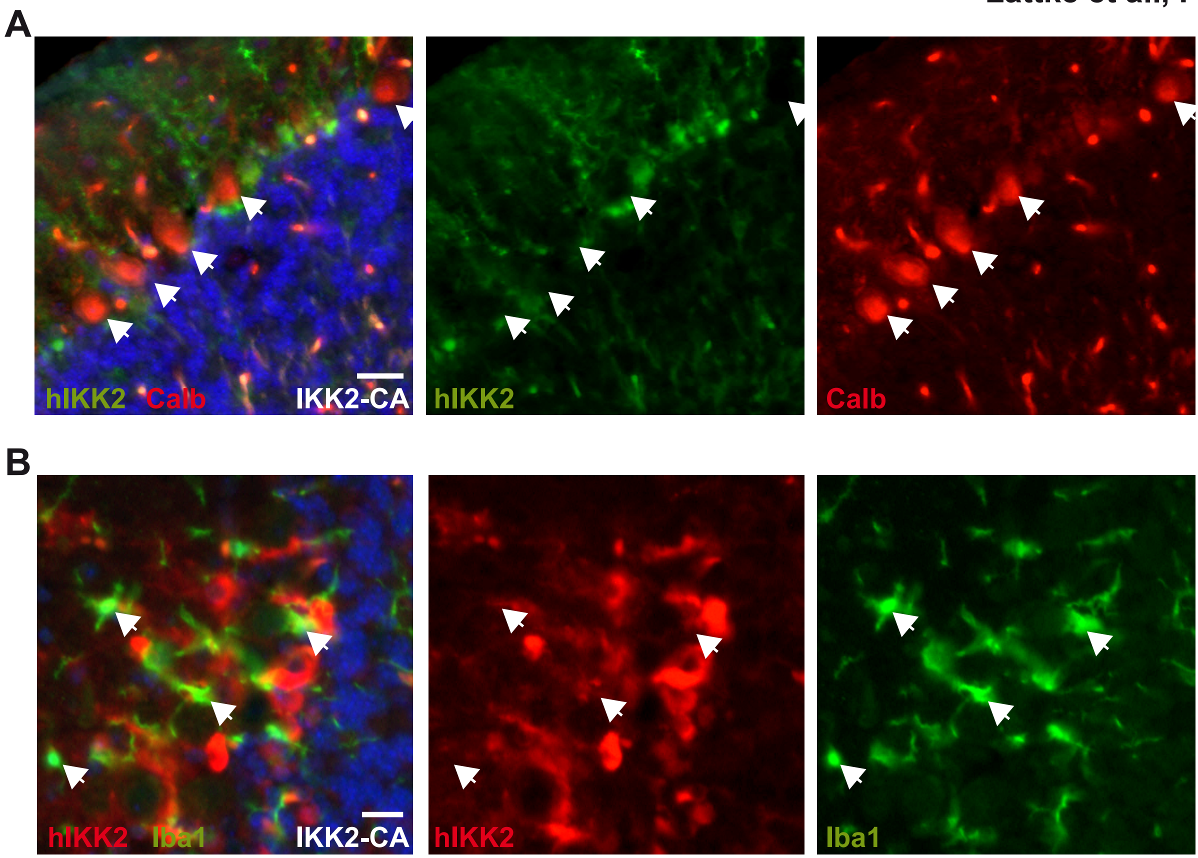
A
hIKK2 Calb
IKK2-CA
hIKK2
Calb
B
hIKK2 Iba1
IKK2-CA
hIKK2
Iba1

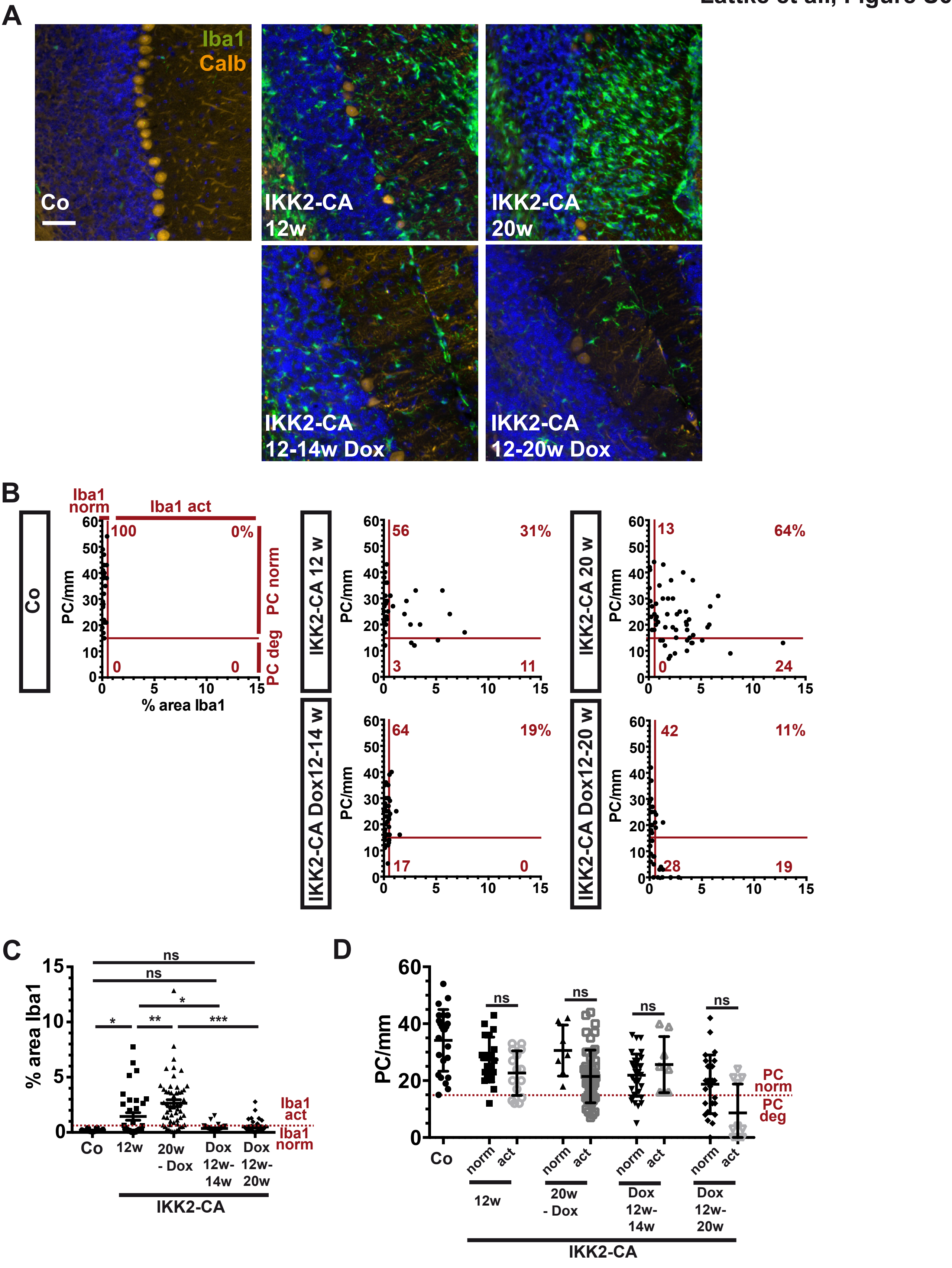
A
Iba1
Calb
Co
IKK2-CA 12w
IKK2-CA 20w
IKK2-CA 12-14w Dox
IKK2-CA 12-20w Dox
B
Iba1 norm
Iba1 act
PC norm
PC deg
Co
PC/mm
% area Iba1
100
0%
0
0
IKK2-CA 12 w
56
31%
3
11
IKK2-CA 20 w
13
64%
0
24
IKK2-CA Dox12-14 w
64
19%
17
0
IKK2-CA Dox12-20 w
42
11%
28
19
C
% area Iba1
ns
ns
*
*
**
***
Iba1 act
Iba1 norm
Co
12w
20w - Dox
Dox 12w-14w
Dox 12w-20w
IKK2-CA
D
PC/mm
ns
ns
ns
ns
PC norm
PC deg
Co
norm
act
12w
20w - Dox
Dox 12w-14w
Dox 12w-20w
IKK2-CA

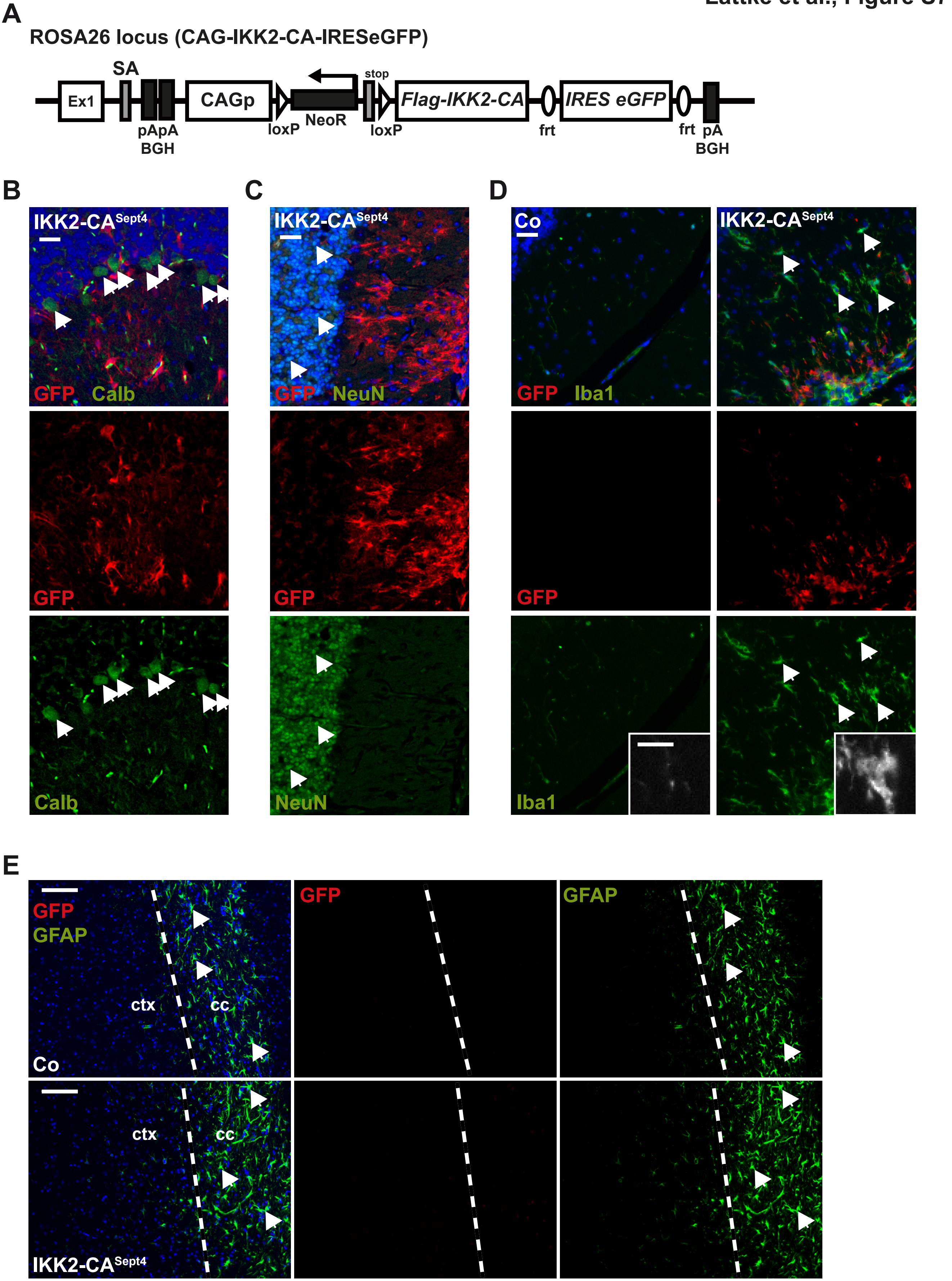
A
ROSA26 locus (CAG-IKK2-CA-IRESeGFP)
SA
Ex1
CAGp
stop
NeoR
Flag-IKK2-CA
IRES eGFP
pApA
BGH
loxP
loxP
frt
frt
pA
BGH
B
IKK2-CA^Sept4
GFP Calb
GFP
Calb
C
IKK2-CA^Sept4
GFP NeuN
GFP
NeuN
D
Co
IKK2-CA^Sept4
GFP Iba1
GFP
Iba1
E
GFP
GFAP
ctx
cc
Co
GFP
GFAP
ctx
cc
IKK2-CA^Sept4

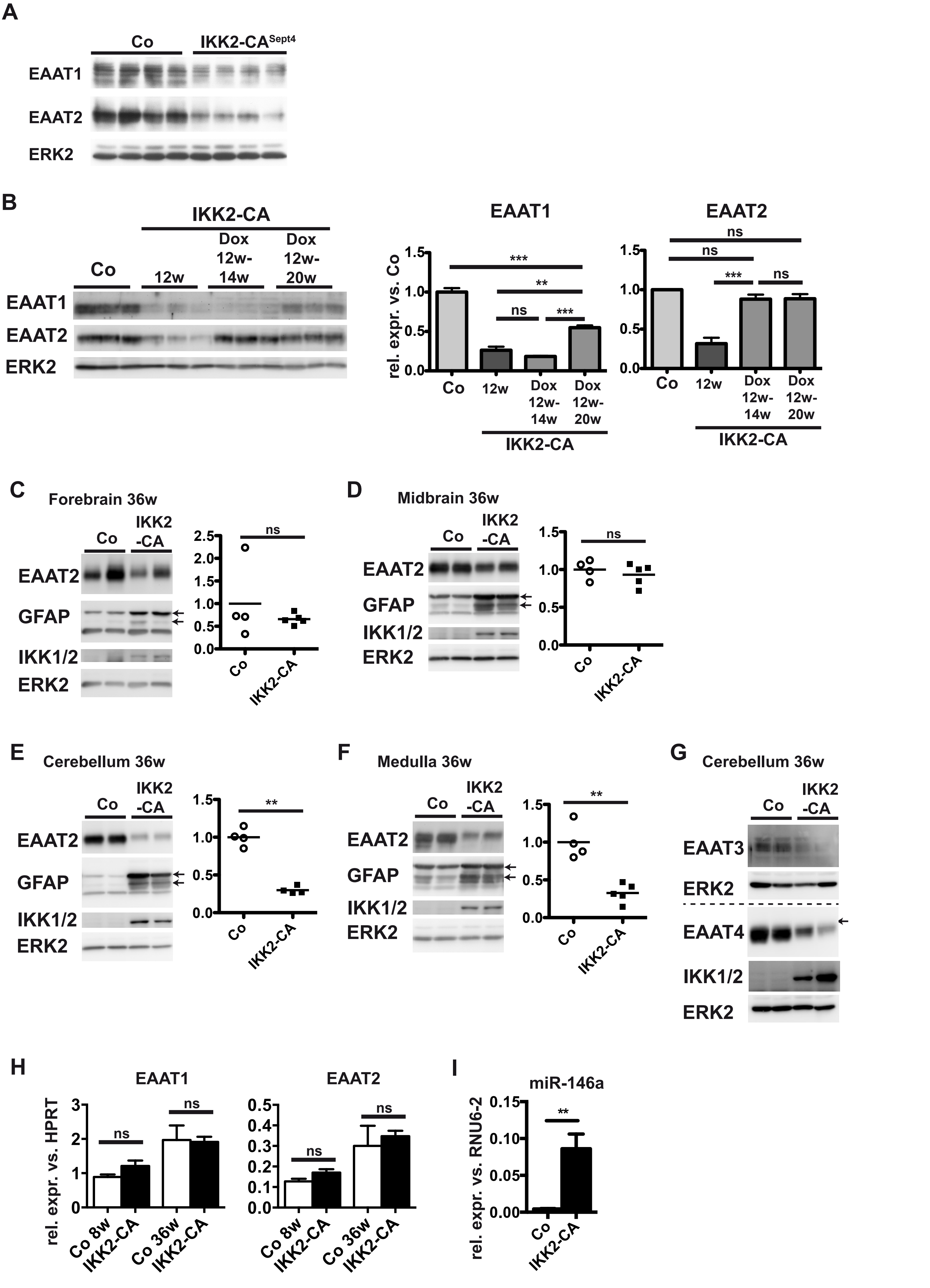
A
Co
IKK2-CA^Sept4
EAAT1
EAAT2
ERK2
B
IKK2-CA
Co
12w
Dox 12w-14w
Dox 12w-20w
EAAT1
EAAT2
ERK2
EAAT1
rel. expr. vs. Co
1.5
1.0
0.5
0.0
***
**
ns
***
Co
12w
Dox 12w-14w
Dox 12w-20w
IKK2-CA
EAAT2
ns
ns
***
ns
Co
12w
Dox 12w-14w
Dox 12w-20w
IKK2-CA
C
Forebrain 36w
Co
IKK2 -CA
EAAT2
GFAP
IKK1/2
ERK2
2.5
2.0
1.5
1.0
0.5
0.0
ns
Co
IKK2-CA
D
Midbrain 36w
Co
IKK2 -CA
EAAT2
GFAP
IKK1/2
ERK2
1.5
1.0
0.5
0.0
ns
Co
IKK2-CA
E
Cerebellum 36w
Co
IKK2 -CA
EAAT2
GFAP
IKK1/2
ERK2
1.5
1.0
0.5
0.0
**
Co
IKK2-CA
F
Medulla 36w
Co
IKK2 -CA
EAAT2
GFAP
IKK1/2
ERK2
1.5
1.0
0.5
0.0
**
Co
IKK2-CA
G
Cerebellum 36w
Co
IKK2 -CA
EAAT3
ERK2
EAAT4
IKK1/2
ERK2
H
EAAT1
rel. expr. vs. HPRT
3
2
1
0
ns
ns
Co 8w
IKK2-CA
Co 36w
IKK2-CA
EAAT2
0.5
0.4
0.3
0.2
0.1
0.0
ns
ns
Co 8w
IKK2-CA
Co 36w
IKK2-CA
I
miR-146a
rel. expr. vs. RNU6-2
0.15
0.10
0.05
0.00
**
Co
IKK2-CA
